# Reinforcement learning for healthcare operations management: methodological framework, recent developments, and future research directions

**DOI:** 10.1007/s10729-025-09699-6

**Published:** 2025-04-09

**Authors:** Qihao Wu, Jiangxue Han, Yimo Yan, Yong-Hong Kuo, Zuo-Jun Max Shen

**Affiliations:** 1 Department of Data and Systems Engineering, The University of Hong Kong, Hong Kong, China; 2 Faculty of Engineering and Business School, The University of Hong Kong, Hong Kong, China; 3 Department of Industrial Engineering & Operations Research, University of California, Berkeley, Berkeley, California USA

**Keywords:** Reinforcement learning, Healthcare operations, Healthcare services delivery, Markov decision process, Approximate dynamic programming, Neural networks

## Abstract

With the advancement in computing power and data science techniques, reinforcement learning (RL) has emerged as a powerful tool for decision-making problems in complex systems. In recent years, the research on RL for healthcare operations has grown rapidly. Especially during the COVID-19 pandemic, RL has played a critical role in optimizing decisions with greater degrees of uncertainty. RL for healthcare applications has been an exciting topic across multiple disciplines, including operations research, operations management, healthcare systems engineering, and data science. This review paper first provides a tutorial on the overall framework of RL, including its key components, training models, and approximators. Then, we present the recent advances of RL in the domain of healthcare operations management (HOM) and analyze the current trends. Our paper concludes by presenting existing challenges and future directions for RL in HOM.

## Introduction

Healthcare operations management (HOM) plays a crucial role in the effective functioning of healthcare systems, impacting public health, patient experience, and healthcare organizational goals. It encompasses various practices that aim to ensure high-quality care, optimize healthcare delivery, and improve overall healthcare outcomes. These practices span a wide range of applications, including patient flow scheduling, medical resources distribution, healthcare process improvement, and pharmaceutical supply chain management [1].

In the field of operations research, traditional approaches to tackling these healthcare operations challenges involve mathematical programming techniques such as mixed-integer programming (MIP) and dynamic programming (DP). These methods formulate the problems as mathematical models and seek optimal solutions by optimizing the specific objective(s). However, as the complexity and scale of these problems increase, conventional mathematical programming approaches may struggle to find optimal solutions due to the *curse of dimensionality* [2].

With the emergence of computing and data science (DS) advancements, an abundance of accessible data and techniques has inundated healthcare operations. Although this presents opportunities for HOM practices, it also poses unprecedented challenges [3]. Mastery of machine learning (ML) and DS techniques is imperative to make good use of such data for better decisions. As a branch of ML, reinforcement learning (RL) optimizes sequential decision-making problems by employing an intelligent agent to determine actions in a dynamic environment. Based on prior experience, the RL agent can learn how to make decisions given the current information, effectively mitigating the curse of dimensionality. RL approaches are developed based on the framework of a Markov decision process (MDP), which is a typical modeling framework in the HOM context. For instance, hospital operations managers determine inpatient admission and discharge on a daily basis given the available information (e.g., patients of different classes in the queues and inpatient beds available) while optimizing multi-objective factors such as health outcomes, operating costs, and benefits. Therefore, RL has gained significant popularity and widespread adoption in HOM.

The application of RL in the healthcare domain presents an attractive opportunity for improving healthcare operations. From a broader perspective, existing surveys on RL in healthcare have discussed various instances across different domains, with a focus on dynamic treatment regimes, interventions, and automated medical diagnosis [4] and clinical decision support [5]. However, a review of RL in HOM is currently not available. Furthermore, the COVID-19 pandemic has severely impacted the global health systems in recent years [6], resulting in various HOM problems due to limited resources such as insufficient COVID-19 test kits for distribution in communities and access block at hospitals [7]. In response, a plethora of RL applications have been employed in corresponding HOM practices. Our paper aims to provide a comprehensive analysis of RL applications in HOM, reviewing the existing methodologies and state-of-the-art solutions. To the best of our knowledge, this review is the first of its kind to analyze RL applications in HOM. Through this review, specific research questions can be answered:

1. Which types of HOM problems hold great potential for deploying RL methods, and how can RL contribute to solving these problems effectively?
2. Which RL methods are most appropriate and effective in addressing specific HOM challenges?
3. What are the main challenges associated with deploying RL in HOM, and how can these challenges be addressed? Furthermore, what are the future directions for RL in HOM, and how can researchers and practitioners leverage RL to enhance healthcare operations?

Our review is structured as follows. In Sect. 2, we offer a tutorial on the RL methodologies that are utilized in healthcare operations, providing a necessary understanding of the required methodologies. Section 3 outlines the scope of our review and describes the methodology employed for the literature search. In Sect. 4, we delve into the specific RL applications within the realm of HOM, classifying them according to their respective scopes and providing detailed analyses of each application. Section 5 summarizes the key insights gained from the reviewed applications and discusses future directions for the utilization of RL in HOM. We highlight potential areas of growth and identify challenges that need to be addressed in order to fully leverage the potential of RL in HOM. Finally, Sect. 6 presents the conclusion that brings together the key findings and contributions of our review.

## Methodologies

This section serves as a tutorial that presents the fundamentals of RL relevant to the applications in HOM. Our tutorial is structured in a manner consistent with other tutorials in different application domains, such as RL for transportation and logistics operations [8, 9].

We start with the essential mathematical framework for modeling sequential decision-making, MDPs [10], which can typically be solved by DP methods using Bellman’s equation [11]. However, classical DP methods may easily fail due to the curse of dimensionality (e.g., in those large-scale or intractable problems). In this case, RL approaches, which are closely related to DP [12], present an alternative class of methods for MDPs and sequential decision-making. Fundamentally, RL algorithms differ from classical DP methods in that they do not always require a complete system model [13]. Hence, they are designed to handle larger-scale problems where traditional exact methods may face computational infeasibility [14]. Nevertheless, RL can also greatly benefit from system models, if applicable, with model-based methods [15].

The RL paradigm is rooted in the vast domain of MDP and sequential decision-making, which often intersects with different subjects and research communities such as operations research (OR), optimal control, computer science (CS), and artificial intelligence (AI). RL is known by various synonyms in different disciplines, such as approximate dynamic programming (ADP) [16], adaptive dynamic programming [17], neuro-dynamic programming [18], heuristic dynamic programming [19], and etc. From the perspective of the OR, researchers usually refer to such approaches as ADP. Powell [16] claimed that ADP is practiced under the umbrella of RL, and also classified classic RL methods (e.g., Q-learning [20]) in ADP. From the perspective of the CS and AI community, RL is a popular terminology, especially with the recent advances in deep learning [21]. The use of MDP in RL (i.e., MDP serves as the environment of RL) has been broadly adopted [12].

In this section, we will walk through certain RL methods for HOM adopted by both the OR and CS communities. Key terminologies in both communities, such as ADP and deep reinforcement learning (DRL), are discussed. Section 2.1 first introduces MDP, which is considered the basic mathematical foundation of RL [12, 21, 22], and briefly discusses classical DP methods (e.g., value iteration [11]). Section 2.2 reviews typical RL methods in HOM, by which the formalized MDP can be optimally solved. Our discussion on RL methodologies begins with standard ADP approaches [16] to classic RL methods (e.g., temporal difference learning [23]), followed by DRL with neural networks. Then, other popular RL techniques, such as policy gradient and bandit problems, are presented [21]. We also intend to present the evolution of RL from classical DP to the recent RL framework that conglomerates multiple aspects. Our paper focuses on RL in HOM, and this section only serves as a high-level tutorial on the RL methods. The reader is recommended to refer to classic references in ADP [16] and RL [21] for more comprehensive discussions.

### Markov decision process

The principle of RL is built upon MDP, which models sequential decision-making problems. The *decision stages* (or *stages*, for simplicity) of the problem are discretized into *T* periods, where $t=0,1,2,\ldots , T$ and *T* is also known as the *horizon* (i.e., the time of termination of the MDP). MDP is typically represented by a tuple $<\mathcal {S}, \mathcal {A}, \mathcal {P}, \mathcal {R}, \gamma>$ [21, 24, 25]. Specifically,

- $\mathcal {S}$ is a set of *states*, and the state at stage *t* is denoted as ${S}_t \in \mathcal {S}$. The state reflects the system’s or environment’s behaviors at every stage via *state variables*. In a finite-horizon MDP, the state sequence is ${S}_0, {S}_1, \ldots , {S}_T$. For the sake of illustrative purposes, this section focuses on finite-horizon MDPs; for MDPs with an infinite horizon, most terminologies and definitions presented here could possibly be generalized for $T \rightarrow \infty$.
- $\mathcal {A}$ is a set of *actions*, and the action at the $t^{th}$ stage is ${A}_t \in \mathcal {A}$. Based on the observed state ${S}_t$ at stage *t*, an action ${A}_t$ is chosen from a set of possible actions $\mathcal {A}$. Similarly, in a finite-horizon MDP, the sequence of actions is denoted as ${A}_0, {A}_1, \ldots , {A}_T$.
- $\mathcal {P}$ is a *transition probability matrix*. Its element, $\mathcal {P}_{s{s'}}^{a}=\mathbb {P}\left( {S}_{t+1} = s' \mid {S}_t = s, {A}_t = a\right)$, measures the probability of transitioning from the current state *s* with action *a* (at stage *t*) to the next state ${s'}$ (at stage $t+1$).
- *R* is a *reward function*, $R({s},{a})=\mathbb {E}[{R}_{t+1} \mid {S}_t =s, {A}_t = a]$. Here, $R_{t+1}$ is the immediate reward generated from the environment given the state-action pair at stage $t+1$. The state-action-reward sequence can be written as ${S}_0, {A}_0, {R}_1, {S}_1, {A}_1, {R}_2, \ldots$.
- $\gamma$ is a *discount factor*, where $\gamma \in [0,1]$. It defines the discounted fraction of a future reward. Specifically, for a reward *r* obtained after $t+1$ stages in the future, its present value would be discounted as $\gamma ^t r$.

An essential characteristic of MDP is that the next state $S_{t+1}$ of the system is only dependent on the current state $S_t$ of the system, and is independent of the history [26], such that $\mathbb {P}[{S}_{t+1} \mid {S}_t]=\mathbb {P}[{S}_{t+1} \mid {S}_1,\ldots ,{S}_t]$.

In MDP, a *policy* $\pi$ defines the rules to choose an action given a state. A deterministic policy maps states to specific actions directly, i.e., adopting exactly the same action in each state. On the other hand, a stochastic policy can be regarded as a mapping from states to probabilities of choosing actions, i.e., ${\pi }(a \mid s)=\mathbb {P}[A_t =a \mid S_t = s]$ [27]. Given policy $\pi$, the *value (state-value) function* $v_{\pi }(s)$, which evaluates the value of being in state *s*, can be written as Eq. 1.

1 $$\begin{aligned} v_\pi (s)\mathrel {\mathop :}=\mathbb {E}_\pi \left[ R_{t+1}+\gamma v_\pi \left( S_{t+1}\right) \mid S_t = s\right] \end{aligned}$$

The *action-value function*, denoted $q_{\pi }(s, a)$, can be written as Eq. 2. The derivations are based on the widely used Bellman’s equation [11].

2 $$\begin{aligned} q_\pi (s, a)&\mathrel {\mathop :}=&\mathbb {E}_\pi [R_{t+1}+\gamma q_\pi \left( S_{t+1}, A_{t+1}\right) \nonumber \\  &   \mid S_t = s, A_t = a] \end{aligned}$$

Eq. 3 presents the objective function of MDP, which aims to maximize the cumulative expected rewards collected over the entire horizon, i.e., 0, 1, 2,..., *T*.

3 $$\begin{aligned} \max _\pi \mathbb {E}_\pi \left[ \sum _{t=0}^{T-1} \gamma ^t R_{t+1} \quad \vline \quad S_0 \right] \end{aligned}$$

To solve this problem, the optimal state-value function $v_*(s)$ and the optimal action-value function $q_*({s},{a})$ need to be identified, and these optimal value functions are associated with an *optimal policy* ${\pi }_*$ [21, 28], which can be determined with Eq. 4.

4 $$\begin{aligned} \pi _*({a} \mid {s})= {\left\{ \begin{array}{ll}1 &  \text{ if } {a}=\underset{ {a}' \in \mathcal {A}}{{\text {argmax}}} \ q_*({s}, {a}'); \\ 0 &  \text{ otherwise } \end{array}\right. } \end{aligned}$$

where $q_*({s},{a})=\max _\pi q_\pi ({s},{a})$ is the *optimal action-value function*. Similarly, $v_*({s})=\max _\pi v_\pi ({s})$ is the optimal *state-value function*.

There is a wide range of real-world problems that can be modeled as MDPs. For example, there were reports of significantly long patient boarding times from emergency departments to inpatient wards [29], especially during the pandemic [7]. In Dai and Shi [30], an MDP framework considering inpatient overflow was proposed for patient boarding decisions. If the primary wards for the patient (i.e., the wards that offer the most effective medical services to the patient) are fully occupied, an overflow policy would allow transfers of patients to other non-primary wards. In this case, the inpatient operations decisions can be determined by an MDP representing a multi-class, multi-pool queuing system. Every state encapsulates information such as patients in each queue at the moment and possible discharges of patients. Given the state, the action of assigning patients to different wards needs to be determined at each decision stage in the planning horizon. This action aims to balance the costs associated with patient overflow and prolonged patient boarding times. After an action is determined, the state transits to the next according to the transition probability matrix.

#### Dynamic programming

When the problem scale is manageable, classical DP methods could be deployed to solve the MDP. *Value iteration* is one of the most widely used approaches [31]. The principle of value iteration is to estimate the value function of each state via an iteration procedure. An initialization procedure (at iteration $n=0$) sets an estimated value of the function, denoted as $v^0 (s)$, to zero. By using the Bellman’s optimality equation [11],

$$ v^{n+1}(s)=\max _{a \in \mathcal {A}} \mathbb {E}\left[ R_{t+1}+\gamma v^n\left( S_{t+1}\right) \mid S_t=s, A_t=a\right] $$

At iteration *n*, the estimated value of the value function, $v^n (s)$, is updated for each $s\in \mathcal {S}$ accordingly [16]:

5 $$\begin{aligned} v^n(s)=\max _{a \in \mathcal {A}}\left( R(s, a)+\gamma \sum _{s^{\prime } \in \mathcal {S}} \mathbb {P}\left( s^{\prime } \mid s, a\right) v^{n-1}\left( s^{\prime }\right) \right) \end{aligned}$$

Value iteration [21] claims the principle of optimality, $v_\pi (s)=v_*(s)$, if and only if $v_\pi (s')=v_*(s')$ for any state *s’* reachable from *s* [28]. Another DP approach, *policy iteration* [21], iteratively alternates between policy evaluation and policy improvement until the policy converges to the optimum.

Such recursive iterations could encounter computational challenges arising from the curse of dimensionality, given that the iterations may need to traverse all the combinations of available states, actions, and transitions [16, 26]. Therefore, methodologies with approximations would be essential to providing practical solutions.

### Reinforcement learning

Based on MDPs, RL approaches could be developed to address these dynamic sequential decision-making challenges. For comprehensive reviews of different classes of RL methods and their applications in various domains, we refer the reader to [8, 9, 32–39]. In this section, we present the fundamentals of RL methods that have been applied in HOM.

#### Approximate dynamic programming

*Approximate dynamic programming* (ADP) is designed for solving large-scale problems and overcoming the “curse of dimensionality" by constructing approximations of value functions (known as *value function approximation*). This subsection introduces how the value function *v*(*s*) of state *s* can be approximated with Monte Carlo sampling. In RL, an *episode* refers to the sequence of agent-environment interactions starting from an initial state and ending in a terminal state, which can be used for learning or evaluating a policy [21]. The principle of Monte Carlo methods is learning from the episodic experience and updating the value estimates based on the average returns observed from episodes [21].

Typically, ADP involves a *rollout process*. In the first phase of ADP, states and possible actions are given to an *approximator* to derive an approximate solution. The states and actions, as in the context of ADP, are usually simpler and of lower dimension than those in the original problem. Once an approximate solution is derived, in the second phase, this approximation is iteratively updated and guides the decision-making process in the original problem [40].

A standard ADP algorithm starts with an initial approximated value function $\overline{V}_t^0(S_t)$ for all states $S_t$ and iteratively updates it in a forward direction based on value iteration (i.e., Eq. 5). At iteration *n*, a *sample path* $\omega ^n$, which refers to a sequence of exogenous information of the system that defines the realizations in all time periods [16], is generated (e.g., by Monte Carlo simulation). Given $\omega ^n$, at any stage *t*, we would be at a realized state $S^n_t$ and need to take an action $a^n_t$. A sampled value $\hat{v}_t^n$ at stage *t* is computed by solving the maximization problem defined in Eq. 6.

6 $$\begin{aligned} {\begin{matrix} &  \hat{v}_t^n= \\ &  \max _{a_t \in \mathcal {A}}\left( R_{t+1}\left( S_t^n, a_t\right) +\gamma \sum _{s^{\prime } \in \mathcal {S}} \mathbb {P}\left( s^{\prime } \mid S_t^n, a_t\right) \overline{V}_{t+1}^{n-1}\left( s^{\prime }\right) \right) \end{matrix}} \end{aligned}$$

where $\overline{V}_t^n (s)$ is the estimated value of the value function at state *s* after *n* sample observations.

From Eq. 6, $a^n_t \in \mathcal {A}$ is set to the optimal action for the maximization problem Eq. 6. Also, $\overline{V}_t^n (s)$ can be updated via Eq. 7:

7 $$\begin{aligned} \overline{V}_t^n\left( S_t\right) = {\left\{ \begin{array}{ll}\hat{v}_t^n, &  S_t=S_t^n \\ \overline{V}_t^{n-1}\left( S_t\right) , &  \text {otherwise}\end{array}\right. } \end{aligned}$$

After $\overline{V}_t^n\left( S_t\right)$ is updated for all $t = 0, 1, 2,..., T$, *n* is advanced to $n+1$. The iteration repeats until it reaches the preset number of iterations *N*.

Finding $\hat{v}_t^n$ and $a^n_t$ via solving the maximization problem Eqs. 6, 7 can be problematic. It could be because the state space is huge and the problem is computationally challenging. Another practical challenge is that the transition function or reward function may not even be known or not mathematically computable [31]. Thus, there are two classes of RL methods that aim to solve the maximization problem Eq. 6: model-based and model-free approaches. *Model-based RL* learns or has access to a model of the environment such that the transition dynamics and reward function can be modeled [41]. On the contrary, without an explicit mathematical model of the environment, *model-free RL* directly learns from experiences or interactions with the environment through trial and error [9].

Researchers from the OR and CS communities may use different names (e.g., “approximate dynamic programming" and “reinforcement learning") to refer to similar RL paradigms [12, 21]. OR researchers typically develop various ADP techniques using mathematically tractable formulations to solve MDPs [42]. On the other hand, CS researchers focus on improving the algorithmic performance of RL methods and approximations in policy space, which will be discussed in subsequent subsections. We consider that the terminology “ADP" emphasizes more on the relationships with the system model, while “RL" emphasizes the approximations by learning [41]. In general, all these RL methods aim to address the challenge of handling high-dimensional problems by using approximations. As healthcare problems have become increasingly large-scale, complex, and dynamic, ADP offers an efficient approach to address various HOM applications [43].

#### Temporal difference learning

*Temporal difference* (TD) learning refers to a popular class of model-free RL algorithms that update the approximations of true value functions (i.e., $v_\pi (s)$ or $q_\pi (s,a)$) based in part on other approximations (i.e., observed samples, for example, $\hat{v}_t^n$) [23]. This general idea is *bootstrapping* [21], and the temporal difference (also known as the Bellman error) is the error in our approximations [16]. TD learning neither requires the episodic outcome nor the complete model of the environment [21].

When approximating the state-value function, the simplest TD method is *one-step TD* that makes the update immediately based on observed $\hat{v}_t^n$, as shown in Eq. 8, where $\alpha$ is the *step-size parameter* [21].

8 $$\begin{aligned} {\begin{matrix} &  \overline{V}_t^n(S_t^n) = (1-\alpha )\overline{V}_t^{n-1}(S_t^n) + \alpha \hat{v}_t^n \\ &  = \overline{V}_t^{n-1}(S_t^n) + \\ &  \alpha \left[ R(S_{t}^n, a_t^n) + \gamma \overline{V}_{t+1}^{n-1}(S_{t+1}^n)-\overline{V}_t^{n-1}(S_t^n)\right] \end{matrix}} \end{aligned}$$

The need for $\alpha$ (i.e., smoothing) arises from the stochastic nature of $\hat{v}_t^n$, which is a consequence of the way employed to estimate the expectation (i.e., model of the environment) [16]. According to sampled exogenous information between *t* and $t+1$ [16], $\hat{v}_t^n=R(S_t^n, a_t^n)+\gamma \overline{V}_{t+1}^{n-1}(S_{t+1}^n)$ is generated on the basis of the transition from $S_{t}^n$ to $S_{t+1}^n$ using a policy (i.e., $a_t^n$) and receiving the reward $R(S_{t}^n, a_t^n)$. In Eq. 8, the term $R(S_{t}^n, a_t^n) + \gamma \overline{V}_{t+1}^{n-1}(S_{t+1}^n)-\overline{V}_t^{n-1}(S_t^n)$ refers to the temporal difference [16].

Approximating the action-value function essentially follows similar approaches for approximating the state-value function previously presented [21]. *TD control* first learns the action-value function rather than the state-value function. TD control can be implemented via *on-policy* or *off-policy* methods. In RL, the policy guiding action selection and subsequent state transition is known as the *behavior policy*, realizing the outcome given the exogenous information. HOM applications usually utilize simulation techniques to generate sufficient sample paths [30, 44, 45]. In the context of simulation, behavior policy could be adopted to control the process of sampling states, which is referred to as *sampling policy* [16]. The policy, which chooses the action that appears to be the best, is referred to as the *target policy* (or also known as the *learning policy*) [16]. On-policy methods, such as *State-Action-Reward-State-Action* (SARSA) [46], improve the target policy that is the same as the sampling policy, whereas off-policy methods improve the target policy that is different from the sampling policy [16, 21].

A typical off-policy method, *Q-learning* (QL) [20] starts with the initialization of action-value function approximation $\overline{Q}_t^0(S_t, a_t)$ for all states $S_t \in \mathcal {S}$ and actions $a_t \in \mathcal {A}$ and iteratively updates the values. At iteration *n*, $a_t^n$ is determined by the sampling policy via Eq. 9:

9 $$\begin{aligned} a_t^n=\underset{a_t \in \mathcal {A}}{\arg \max } \overline{Q}_t^{n-1}\left( S_t^n, a_t\right) \end{aligned}$$

Then, $\overline{Q}_t^n(S_t^n, a_t^n)$ is updated via Eqs. 10 and 11:

10 $$\begin{aligned} \hat{q}_t^n = R\left( S_t^n, a_t^n\right) + \gamma \max _{a' \in \mathcal {A}} \overline{Q}_{t+1}^{n-1}\left( S_{t+1}^n, a'\right) \end{aligned}$$

11 $$\begin{aligned} {\begin{matrix} &  \overline{Q}_t^n\left( S_t^n, a_t^n\right) = (1-\alpha ) \overline{Q}_t^{n-1}\left( S_t^n, a_t^n\right) + \alpha \hat{q}_t^n \\ &  = \overline{Q}_t^{n-1}\left( S_t^n, a_t^n\right) + \alpha [R\left( S_t^n, a_t^n\right) \\ &  + \gamma \max _{a' \in \mathcal {A}} \overline{Q}_{t+1}^{n-1}\left( S_{t+1}^n, a'\right) -\overline{Q}_t^{n-1}\left( S_t^n, a_t^n\right) ] \end{matrix}} \end{aligned}$$

Then, the transition to $S_{t+1}^n$ and the reward $R(S_t^n, a_t^n)$ are obtained based on the exogenous system information (e.g., from a sample path) observed at stage *t*. In Eq. 10, QL (off-policy) includes a maximization problem $\max _{a' \in \mathcal {A}} \overline{Q}_{t+1}^{n-1}\left( S_{t+1}^n, a'\right)$ to select an action for the update. Instead, SARSA (on-policy) replaces this problem with $\overline{Q}_{t+1}^{n-1}(S_{t+1}^{n}, a_{t+1}^{n})$, where $a_{t+1}^{n}$ is generated following the same policy that determines $a_t^{n}$ (i.e., in Eq. 9) [16].

Given a set of approximated Q (action-value) functions $\overline{Q}^n(s, a)$, the approximated state-value function can be computed using Eq. 12 [16].

12 $$\begin{aligned} \overline{V}^n(s)=\max _{a \in \mathcal {A}} \overline{Q}^n(s, a) \end{aligned}$$

In this way, Eq. 10 can be reformulated as Eq. 13.

13 $$\begin{aligned} \hat{q}_t^n=R\left( S_t^n, a_t^n\right) +\gamma \overline{V}_{t+1}^{n-1}\left( S_{t+1}^{n}\right) \end{aligned}$$

When comparing $\hat{q}_t^n$ in Eq. 13 with $\hat{v}_t^n$ in Eq. 6, the embedded expectation over the downstream states that arise from action $a_t$ have to be calculated to identify $\hat{v}_t^n$ in Eq. 6; however, this step is always not computational efficiency. On the contrary, QL takes an action following the sampling policy and observes the downstream state given the exogenous information. In HOM studies that feature finite and discrete actions, QL addresses problems that traditional DP can hardly resolve, such as routing problems in rescuing and emergency services [47–50].

TD methods leverage the advantages of the use of bootstrapping in DP and the sampling capabilities of Monte Carlo simulation [21]. TD learning methods can be unified as TD($\lambda$) according to the use of *eligibility traces* [23], $\lambda \in [0,1]$, which represent the algorithmic discount to control the weights for expected rewards from different decision stages [21]. For example, the a higher value of $\lambda$ leads to greater weights of rewards that are from distant states and actions. TD(0) (i.e., one-step TD [21]) uses one future reward $R\left( S_t^n, a_t^n\right)$ to update the value function approximation (Eq. 8), while TD(1) implements a Monte Carlo algorithm [28] that updates the value function approximation using episodic outcomes [21].

#### Value function approximation

This subsection discusses several popular approximate solution methods in HOM. In basic settings of ADP and TD methods (as discussed in Sects. 2.2.1 and 2.2.2), the lookup table plays the role of approximator for value function approximations [16, 21]. For example, the Q table of QL records the values for each visited state-action pair in a tabular form during the iterations based on samples [12]. Therefore, algorithm performance may still be constrained by the sizes of states and actions [16, 21]. Fortunately, there are various kinds of function approximations rather than tables to address the curse of dimensionality in state space better.

In the context of ADP, a *basis function* $\phi _f(S_t)$ maps state information from $S_t$ to a value of feature *f* by approximation [16, 31], where $f \in \mathcal {F}$ is a feature in the feature set $\mathcal {F}$. In this way, the approximators of ADP could be constructed using a set of operators and transformation techniques, including lookup tables, aggregation, linear regression, kernel regression, and polynomial regression. For example, linear value function approximation $\overline{V}_{\varvec{\theta }}(S_t)$ (which is a parametric model [16]) with approximators’ parameter vector $\varvec{\theta }$ can be written in Eq. 14:

14 $$\begin{aligned} \overline{V}_{\varvec{\theta }}(S_t)=\sum _{f \in \mathcal {F}} \theta _f \phi _f(S_t) \end{aligned}$$

Recently, RL using nonparametric models [16], such as neural networks, as approximators for value function approximation has drawn growing attention. As an instance of supervised learning, function approximation generalizes from samples of a desired function (e.g., value function) to formulate an approximate representation of the entire function [21]. Those typical algorithms in HOM are studied in the following subsections.

#### Deep Q-network

Generally speaking, *deep Q-network* is similar to QL but uses neural networks to approximate the value function, rather than the QL’s tabular method. RL methods with function approximation by deep artificial neural networks are considered *deep reinforcement learning* (DRL) [21]. These *deep neural networks* (DNN) include *multi-layer perception* [51], *convolutional neural networks* (CNN) [52], and *recurrent neural networks* (RNN) [53]. We refer the reader to [54] for an inspiring discussion on neural networks and deep learning [54]. Our study suggests that several renowned models, such as *long short-term memory* (LSTM) [55], *graph neural network* (GNN) [56], *transformer*, and *attention mechanisms* [57], have been successfully employed as approximators of reinforcement learning in HOM practices.

*Deep Q-network* (DQN) is a widely applied DRL method that has been successfully applied in various industries. It has even achieved human-level control in Atari video games [58, 59]. The primary principle of DQN is to replace the Q table approximator in Q-learning with neural networks. In each decision stage, the state variables are fed into the DQN neural networks (referred to as *Q-networks*), which compute the approximated action-value function. The optimal action is then chosen by solving a maximization problem similar to Eq. 9.

A key component of the DQN method is the use of *experience replay* [59]. This technique involves storing the agent’s experiences, represented as transitions $e_t=(s_t, a_t, r_{t+1}, s_{t+1})$, to a dataset $D_t=\{e_1,\ldots ,e_t\}$ at each stage *t*. During the learning process, DQN performs Q-learning updates on batches of experience samples $e=(s, a, r, s') \sim U(D)$, where *U*(*D*) denotes a uniform random sampling from the stored transitions [59]. After an action is chosen, the DQN agent stores the newly generated transitions to the dataset. Another important aspect of DQN is the concept of *fixed Q-targets* [59]. This mechanism controls the frequency at which the parameters $\varvec{\theta }$ of the Q-networks are updated. At predefined intervals, the *target Q-network* (also known as the *fixed Q-network*, which approximates the target Q-function $\overline{Q}_{\varvec{\theta }}$, is synchronized with the latest parameters $\varvec{\theta '}$ of the current Q-network. The current Q-network is used for choosing the optimal action by approximating the current Q-function $\overline{Q}_{\varvec{\theta '}}$ during sampling or making the decision (as shown in Eq. 9).

DQN is a model-free off-policy method [58]. The loss function utilized in the *i*-th update is as follows.

15 $$\begin{aligned} L_i(\varvec{{\theta _i}})= \underset{e \sim U(D)}{\mathbb {E}}\left[ (r + \gamma \max _{a' \in \mathcal {A}} \overline{Q}_{\varvec{\theta _i}}(s', a') - \overline{Q}_{\varvec{\theta _i'}}(s, a))^2\right] \end{aligned}$$

In Eq. 15, the DQN agent computes the *target* *Q*-*value*, $r + \gamma \max _{a' \in \mathcal {A}} \overline{Q}_{\varvec{\theta _i}}(s', a')$, of the batched samples $e \sim U(D)$ based on the target Q-network. Subsequently, *stochastic gradient descent* is implemented to minimize this loss function with respect to the parameter $\varvec{\theta _i'}$. The experience replay and fixed Q-targets are designed to avoid autocorrelation and ensure the learning quality [59].

Several variants of DQN have been developed to address issues such as overestimation and difficulties in convergence. One such variant is Double DQN (DDQN) [60]. DDQN uses two function approximators: one to select the optimal actions and another to compute the target Q-value. The target Q-value is computed as $r + \gamma \overline{Q}_{\varvec{\theta _i}}\left( s', \underset{a' \in \mathcal {A}}{{\text {argmax}}} \ \overline{Q}_{\varvec{\theta _i'}}(s', a')\right)$. Using two separate function approximators, DDQN reduces the overestimation of action values and improves learning performance [60].

Dueling DQN takes a different approach to constructing the target Q-value by summing the state-value function and the actions’ *advantage function* [61]. The advantage function has a size equal to the action space. The idea is to decompose the estimations of state and action, so as to improve learning convergence and performance. Dueling DDQN (D3QN) integrates the techniques of DQN, Double DQN, and Dueling DQN. This combination of methods has been shown to offer an effective decision-making approach in various domains, including transport and healthcare [62–64].

Compared with QL, the DQN family is capable of handling HOM problems with larger state spaces because of using neural networks for generalization in function approximation. Hence, more complex routing problems in healthcare logistics [65] and supply chain [66] could be optimized.

#### Policy gradient

Unlike value-based methods (e.g., ADP, QL, and DQN) that update optimal policies according to approximated value functions, *policy gradient* [67] directly optimizes the *policy objective function* $\mathcal {J}(\varvec{\vartheta })$ with respect to its policy’s parameter $\varvec{\vartheta }$, and determines actions based on the approximated probability distributions. Such an approach enables policy gradient to implement both discrete and continuous actions. In order to maximize the performance of the policy, the gradient of the value function with respect to the policy parameters, $\nabla \mathcal {J}(\varvec{\vartheta })$, is utilized, as shown in Eq. 16 [21]:

16 $$\begin{aligned} \nabla \mathcal {J}(\varvec{\vartheta }) = \mathbb {E}_\pi \left[ \sum _{a \in \mathcal {A}} q_\pi \left( S_t, a\right) \nabla \pi \left( a \mid S_t, \varvec{\vartheta }\right) \right] \end{aligned}$$

As a Monte Carlo method, the direct use of the typical *REINFORCE* (policy gradient) algorithm [68] updates policies with the entire episode of transitions and return, while suffering from the large variance and slow learning [21]. *Actor-critic* (AC) algorithms, a class of model-free policy gradient RL methods that leverage the strengths of both policy-based and value-based approaches [69, 70], can substantially reduce variance in learning. AC consists of two approximators: the *actor* (which determines the policy $\pi _{\varvec{\vartheta }}(s, a) = \pi ({a} \mid {s}, \varvec{\vartheta })$) and the *critic* (which estimates the value function $\overline{Q}_{\varvec{\theta }}(s, a)$). Here, $\varvec{\vartheta }$ and $\varvec{\theta }$ are the respective parameters in actor and critic neural networks. AC’s approximate policy gradient can be formulated as Eq. 17 [28]:

17 $$\begin{aligned} \nabla _{\varvec{\vartheta }}\mathcal {J}(\varvec{\vartheta }) \approx \mathbb {E}_{\pi _{\varvec{\vartheta }}}\left[ \nabla _{\varvec{\vartheta }}\log \pi _{\varvec{\vartheta }}(s, a) \overline{Q}_{\varvec{\theta }}(s, a) \right] \end{aligned}$$

In Eq. 17, $\overline{Q}_{\varvec{\theta }}(s, a)$ approximated by the critic neural network can also be replaced by the advantage function, which measures the relative advantage of taking action *a* in state *s* over the average. The advantage function efficiently reduces the variance of policy updates. The AC algorithm with the advantage function is known as *advantage actor-critic* (A2C) [71], which can be extended to *asynchronous advantage actor-critic* (A3C) [71, 72] with parallel computations for multiple agents’ interactions. These algorithms have also been widely adopted in HOM, such as hospital expansions [73, 74] and inventory control [75], in which the curse of dimensionality in both state and action spaces (e.g., determining the production and transportation capacities of regenerative medicine [75]) could be effectively addressed.

*Proximal policy optimization* (PPO) [76] is a widely used on-policy algorithm that builds on AC. PPO aims to address the instability and sensitivity issues associated with vanilla policy gradient methods [77]. PPO enforces a constraint on the policy update to ensure that the new policy does not deviate too much from the old policy. This is achieved by introducing a clipped surrogate objective function that leverages the advantage function [78] and Kullback–Leibler (KL) divergence [79].

*Deep deterministic policy gradient* (DDPG) is a model-free, off-policy RL algorithm that combines the strengths of DQN [58] with deterministic policy gradients [80, 81]. DDPG adopts the AC architecture, where the actor (neural network) learns a deterministic policy $\pi _{\varvec{\vartheta }}(s)$ that maps states to actions, and the critic (neural network) learns the function $\overline{Q}_{\varvec{\theta }}(s, a)$ that maps state-action pairs to values. Here, $\varvec{\vartheta }$ and $\varvec{\theta }$ are the parameters of the actor and critic neural networks, respectively. The objective is to maximize the expectation as shown in Eq. 18; the utilization of experience replay, $e \sim U(D)$, is the same as in Eq. 15 for DQN.

18 $$\begin{aligned} \max _{\varvec{\vartheta }} \underset{e \sim U(D)}{\mathbb {E}}\left[ \overline{Q}_{\varvec{\theta }}\left( s, \pi _{\varvec{\vartheta }}(s)\right) \right] \end{aligned}$$

DDPG incorporates two key techniques, experience replay, and fixed Q-targets, similar to those used in DQN [80, 81]. These techniques are employed to enhance sample efficiency and stabilize the training process.

The policy gradient algorithms mentioned above have been successfully applied in HOM, with many falling under the AC algorithm family. These algorithms were selected due to their robustness, ability to handle continuous action spaces, and high sample efficiency, which are crucial in healthcare settings [5].

#### Exploration and exploitation

In RL, algorithms are required to overcome the *exploration-exploitation trade-off* dilemma [21] when optimizing their decision policies. This trade-off arises from the need to balance between exploring uncertain actions to gain new knowledge about the system, such as the probability distributions of rewards, and exploiting the best actions given current already-known knowledge, in order to maximize the long-term rewards.

The exploration-exploitation trade-off is exemplified by the *multi-armed bandit* (MAB) problem [82, 83], in which the agent is likened to a gambler who must choose “one arm of the bandit" from multiple options, each with unknown reward probabilities. The MAB problem is typically considered in a special case of RL, which has a single-state environment and immediate rewards. This setting makes the required solution procedures more computationally efficient. Its goal is to maximize the cumulative rewards obtained [21]. According to [84], there exist several bandit strategies that can be used to determine optimal actions. While the *epsilon-greedy* strategy [21] is widely used, other approaches such as *upper confidence bounds* [85], *Thompson sampling* [86], and *Gittins index* [87] have also been applied in the literature of HOM. With an upper confidence bound approach, the action with the highest reward is chosen, while Thompson sampling (rooted in Bayesian methods) selects actions based on their posterior probabilities of being the best [21]. These algorithms are widely applied in resource allocations in HOM, such as vaccine allocation [88–90] and outpatient management [91, 92], given their strong interpretability, sound theoretical support, and adaptivity in dynamic environments.

Further, *Bayesian RL* [93] is designed to address the exploration-exploitation trade-off. By leveraging the *prior* probability distribution that represents uncertainty over value function approximations, Bayesian RL incorporates *Bayesian inference* to update the prior and obtain a *posterior distribution* based on observed transitions [94]. This approach allows the learning agent to explicitly incorporate uncertainty by treating the states of the MDP as hyper-states [95] when making decisions. This integration of uncertainty with the states enables more effective exploration strategies. The exploration-exploitation trade-off is naturally considered in Bayesian RL as the transitions occur among different hyper-states that involve uncertainty [9]. In this framework, Bayesian inference can serve as an approximator, and the knowledge about the prior distribution becomes more important [96].

#### Learning complex systems

*Multi-agent reinforcement learning* (MARL) [97] extends RL to handle HOM problems in more complex systems by involving multiple decision-making agents. In MARL, each agent learns its own local policy, and these individual policies are utilized to form a joint policy that maximizes the overall expected reward [98]. The interactions among multiple agents can vary from cooperative settings to dynamic competitive games. For the details, we refer the reader to surveys on multi-agent systems [99] and the Markov games framework [100] of MARL. In this way, the curse of dimensionality in complex HOM problems, such as coordinating multiple emergency departments [101] or emergency vehicles [102], could be addressed.

*Hierarchical RL* [103] is a solution method that aims to solve complex problems efficiently by breaking them down into simpler structured subproblems. This approach involves organizing the problem into multiple levels of abstraction, each with its own set of policies. At the high level, there are policies (also known as *options*) [104], which make decisions less frequently and focus on broader objectives, similar to the functions of managers. On the other hand, low-level policies are responsible for implementing immediate and finer-grained actions in the environment, similar to the functions of workers. This hierarchical approach is particularly effective in handling tasks with large state and action spaces, such as human–machine collaboration in ventilator production [105], as well as environments that provide sparse rewards [106].

*Imitation learning* [107], a methodology tailored for complex systems, involves recovering the reward function from expert demonstrations through the theory of *inverse RL* [108]. Rather than relying solely on trial and error, an imitation learning agent can swiftly adopt decision-making policies from established human experts’ policies [107]. HOM utilizes *behavioural cloning* [109], an imitation learning technique that trains the RL agent to replicate the policies of experts based on the observed states, a process that parallels supervised learning [110].

Furthermore, RL has been employed as an optimizer within complex algorithms, such as those used for predicting healthcare-related metrics during epidemics, including numbers of infections and inpatient admissions [74, 111–113]. In these scenarios, RL not only enhances prediction accuracy by optimizing the hyperparameters of the student-teacher curriculum learning [111], but it also identifies the key features that influence the system [114]. While heuristic methods [115] can accomplish similar optimization tasks, a more promising approach lies in combining RL with heuristics to leverage their complementary strengths in combinatorial optimization [116, 117].

### Summary of key RL settings

Based on our previous discussion, we summarize the key RL settings in the context of HOM applications.

#### Model-based versus model-free

Model-based RL makes use of the system model. Thus, the transitions and reward function could be explicitly incorporated into the solution framework [41]. For example, in inpatient management [30], model-based RL could leverage the queuing network to infer value functions from waiting and overflow costs, by which the optimized policies could be built on analytical properties. However, model-free RL does not utilize the system model; rather, it learns directly from empirical interactions within the environments’ simulations using an iterative trial-and-error approach. It heavily relies on training samples and may suffer from poor sample complexity and convergence issues. Generally, leveraging domain knowledge and problem structure can accelerate convergence and reduce computational time for model-free methods [105, 118].

#### Tabular versus non-tabular

Referring to Sect. 2.2.3, value function approximation can be classified into *tabular* and *non-tabular* approaches. In problems consisting of only small numbers of states and actions, approximation could be completed with tabular methods. In the forms of arrays or tables, each row/column is associated with a state or state-action pair. Standard ADP and TD learning utilize tabular approximations that are derivative-free [31]. However, in many HOM practices, the huge number of states may impose computational challenges in utilizing tabular approaches. In these cases, RL methods using more compact and non-tabular forms of function representation are needed [21].

#### Value-based versus policy-based

As we have systematically introduced in Sect. 2.2.2, given state *s* (or state-action pair (*s*, *a*)), *value-based* approximation estimates $v_{\pi }(s)$ (or $q_{\pi }(s,a)$) through value function approximation. The optimal policy (Eq. 4) is approximated by iteratively updating the approximated Q-value ($\bar{Q}(s,a)$) in Eq. 9. Value-based methods typically require explicit computations for each action. Therefore, some studies on pandemic control [119–121] considered discretized thresholds to represent lockdown policies based on state variables. Referring to Sect. 2.2.5, *policy-based* approximation parameterizes and determines the policy without using value functions. It requires differentiability of the policy $\pi (a \mid s, \varvec{\vartheta })$ to determine the parameter $\varvec{\vartheta }$, so as to avoid solving the potentially intractable maximization problem in Eq. 4. In this way, policy-based methods can handle continuous action spaces. Stochastic policies are favored in policy-based methods due to their differentiability. In many situations, policy-based methods can be combined with value-based methods, such as the AC algorithms, to reduce variance in updates [21].

#### On-policy versus off-policy

In Sect. 2.2.2, we discuss that on-policy methods focus on assessing or enhancing the policy that dictates decision-making, while off-policy methods aim to evaluate or refine a policy distinct from the one utilized to generate the data [21]. Thus, on-policy methods are relatively simple and stable in their learning. Off-policy methods are flexible in learning a broader range of data, such as human experiences in HOM, but suffer from greater variance and slow convergence [21]. Given that simulations can mitigate the lack of samples in HOM, we have observed extensive applications of both on-policy and off-policy methods in the following Sect. 4.

#### Online learning versus offline learning

Online learning continuously updates approximations’ parameters as new data (e.g., states, actions, and rewards) arrive without re-training from scratch. It is particularly suitable for dynamic environments where adaptability is essential, such as HOM applications [88, 92, 122]. *Bandit problem* [16] (in Sect. 2.2.6), focusing on single-step decisions with partial feedback, is a specific subclass of online learning. On the other hand, offline learning updates approximations’ parameters according to the fixed entire dataset that is available at the time of training. This process may iterate several rounds until approximations’ performance stably achieves defined criteria. Some offline methods with experience replay [59] are discussed in Sects. 2.2.4 and 2.2.5. One limitation of offline learning is that storing the entire training set may cause memory issues (from the computational resource point of view), especially when setting a large batch size or the state space has to be huge to describe HOM problems (e.g., in pandemic control application [121]).

## Review scope and search

Our work adopts a scoping review approach [123] to review and analyze relevant research studies. We focus on HOM applications rather than clinical diagnostics; thus, publications in precision medicine development, medical imaging, and medical robotics are excluded. Following the healthcare ecosystem map [124], we are able to identify keywords that are closely associated with our HOM scope. These keywords include “healthcare", “operations management", “hospital", “patient", “medical", “public health", “epidemic", “pandemic", “emergency", and “humanitarian". We implemented a search strategy that contained a certain word “reinforcement learning" followed by these keywords on *Scopus*, and limited the subject area to “Decision Science". The Scopus query syntax is TITLE-ABS-KEY ( “reinforcement learning" ) AND TITLE-ABS-KEY ( “healthcare" OR “operations management" OR “hospital" OR “patient" OR “medical" OR “public health" OR “epidemic" OR “pandemic" OR “emergency" OR “humanitarian" ) AND ( LIMIT-TO ( SUBJAREA, "DECI" ) ). This initial search of articles (conducted on January 4, 2023, and updated on January 22, 2024) resulted in 321 documents. Based on our knowledge, we included additional relevant articles (e.g., those in *arXiv* and conference proceedings) since RL is also a widely researched area within the computer science community. After an initial checking of the abstracts, we considered a total of 144 articles for further analysis. In the subsequent round of detailed content analysis, we identified 117 relevant studies on RL in HOM for our review.

## Applications

Following [124], we categorize the studies into *macrolevel*, *mesolevel*, and *microlevel* research thrusts. We also adopt the terminologies, classification, and empirical results from previous studies such as [125] and [126]. We consider the healthcare ecosystem map presented in [124] to structure our three thrusts of healthcare operations applications. The macrolevel research thrusts entail the overarching strategy and policy implemented by governments or authorities to harness the healthcare marketplace. The mesolevel research thrusts serve as a connector between the macrolevel and microlevel research thrusts. For example, it encompasses the distribution and allocation of resources across multiple healthcare facilities. Finally, the microlevel research thrusts pertain to the detailed operations involved in providing patient care services within a healthcare facility.

### Macrolevel research thrusts

Following the discussion in the literature [124, 127, 128], the applications in the macrolevel research thrusts revolve around the supply of and demand for healthcare services through various healthcare entities (e.g., hospitals, pharmacies, and governments) and on marketplaces. Examples include market mechanisms, organizational structures, healthcare network flows, and accessibility to health services. We analyze the relevant RL applications and identify that the majority of such applications focused on healthcare policies and strategies. A portion of these RL applications was studied by Weltz et al. [129] with a specific focus on respondent-driven sampling in public health, leaving a comprehensive review yet to be conducted. The global outbreak of COVID-19 has led to a surge in recent research focused on utilizing RL to determine optimal pandemic intervention policies. Interestingly, we find that the RL studies in the macrolevel research thrust focus on infection modeling and control. We classify the studies into general measures and strategies, COVID-19 control policies, and mobility restriction policies.

#### General measures and strategies

Prior to the outbreak of COVID-19, there were already research studies on sequential decision-making in public health, ranging from model-based simulation to ADP and DQN. Back in 2008, Das et al. [130] published a research study that developed a simulation model for analyzing large-scale pandemic outbreaks to minimize the aggregated costs resulting from healthcare expenses and lost wages. Their study considered community, demographic, physiological, behavioral, and epidemiological features, such as indicators of infection, the stockpile of vaccines and drugs, as well as the hospital capacity. The decisions for the considered mitigation strategies encompass a range of actions and plans, including prophylaxis, quarantine plans, and hospital admissions. RL was also proposed as a solution to the problem. In a more recent study, Shi et al. [132] conducted simulations on voluntary vaccination in social network settings and found that heterogeneous social connections demonstrate greater sensitivity to information regarding vaccination. These simulations were specifically designed for an RL environment.

Regarding school closure and vaccinated cohorts for controlling the H1N1 epidemic [151], Yaesoubi et al. [131] adopted a partially observable Markov decision process (POMDP) [152] in modeling hospitalizations and vaccinations. The study concluded that an ADP approach guided by the latest information outperformed static policies. Their results highlighted the significance of incorporating real-time data into decision-making processes. Probert et al. [133] applied DQN to contain outbreaks of foot-and-mouth disease in farms using a Susceptible-Exposed-Infectious-Recovered (SEIR) model [153]. Their approach modeled the RL state (e.g., infected and susceptible farms) on a discretized landscape, with a CNN serving as the approximator. The state-dependent actions involve selecting which farms to cull.

In a recent study, Liu et al. [134] developed an approach for adaptive control of the Ebola virus disease spreading across multiple locations. They utilized a combination of deep spatial fitted Q iteration [154] with graph embeddings (a GNN approach), a semi-parametric variant [155] of Thompson sampling, and a tractable quadratic program [156] to handle the search in a large action space. Comparisons with ad-hoc strategies and a susceptible-infected-susceptible (SIS) [157] model-based policy search showed that their proposed method achieved better control (resulting in more disease-free individuals) and higher robustness to model misspecification. They also provided insightful discussions on the topics of causal inference [158] and interpretability [159] of the RL solutions.

#### COVID-19 control policies

In late 2019, the COVID-19 pandemic broke out, quickly spreading worldwide and impacting billions of individuals. To address the unprecedented challenges posed by the COVID-19 pandemic, researchers have explored the application of RL in developing intervention policies and devising healthcare strategies. These strategies include testing, sanitization, and lockdown measures [135].

By using SEIR models and DQN, Arango et al. [136] and Miralles et al. [137] determined optimal lockdown policies to optimize the number of available beds in intensive care units (ICUs) and the economic costs. Only infections were considered the state variable, while other variables depended on it. This approach aimed to approximate the disease transmission rate based on the number of infections. Their studies also suggested short lockdown cycles as solutions. In a later study, Padmanabhan et al. [140] developed QL approaches to implement closed-loop control by sequentially determining intervention actions in Qatar.

From a perspective of Bayesian inference, Rathore et al. [141] proposed both Bayesian RL and control theory to reduce the impacts of respiratory infectious pandemics (such as COVID-19). They utilized a susceptible-infectious-recovered (SIR) model and POMDP to study the infectious disease outbreak. In the pandemic process, three states – pre-trigger, increasing, and decreasing – were considered. The authors introduced a control knob represented by the reproduction number to indicate the on-off signals of actions. This approach enabled the RL agent to leverage pre-trigger policies in an offline manner initially. The policies were then transferred to an online exploration approach based on the information state and its associated likelihood. Wan et al. [120] developed an adaptive MARL approach to identify Pareto-optimal policies. They established a Bayesian epidemiological model with online learning. They employed a delayed MDP framework to generate a proxy state to capture the time-lag relationships between the number of infected and confirmed cases. In addition to DQN, they utilized Monte Carlo rollouts that considered real-life constraints, such as the severity of the spread, enhancing the interpretability of the results. Their experiments suggested that these robust methods could effectively control epidemics in various cities with reduced costs.

Another line of research studies the impacts of pandemic control policies at an individual level. Several studies have incorporated weighted rewards to account for economic impacts and infections at an individual level. Ohi et al. [119] utilized LSTM and DDQN to determine optimal epidemic control policies for three levels of restriction policies. Based on population density and reproduction rates, they proposed placing a long lockdown during the first surge, followed by cyclic and short lockdowns to prevent resurgence. Khadilkar et al. [138] factored in individual costs and developed a propagation model using network data. Using DQN, their proposed policy resulted in a higher peak of infections but a shorter epidemic lockdown duration than a static threshold policy. Kompella et al. [139, 160] extended the SEIR model by incorporating more detailed components related to locations, testing and tracing, and government regulations in their proposed AC approach. Their proposed method considered partially observed states capturing aggregated testing results and the number of hospitalizations. Their results suggested stratified actions consisting of combinations of government regulations. Their experiments were scaled up to a population of 10,000 individuals while ensuring that actions were stable. However, the computational expense of the proposed approach may pose a challenge when scaling up to a national epidemic control scenario at the macrolevel.

To date, the COVID-19 pandemic has presented an impetus for scholars and researchers to delve into the utilization of RL in the formulation and implementation of macrolevel healthcare policies. The existing studies have demonstrated that policies derived from RL approaches provide more cost-effective solutions [143, 144] than relying on heuristics or expert opinions when balancing saving lives and reducing economic impacts. Guo et al. [121] built upon previous works such as DQN [137] and agent-based FluTE simulation [161]. They expanded the established state variables, including vaccinations, the net monetary impacts of pandemic severity, and lockdown policy (strictness of the policy). A ProbSparse self-attention mechanism [162] was integrated into the perceptron model to extract crucial information from complex epidemiological observations. This fusion facilitates the effective processing of high-dimensional data in the context of epidemiology. Bushaj et al. [142] emphasized the importance of increasing the number of healthy individuals in a population, early random vaccination of potential super spreaders, and quarantining high-risk individuals. They extended the Covasim simulation model [163] by implementing random and age-based vaccination strategies. They integrated compartmental information, such as the population with the two-shot vaccine, into the state space of their DQN. Additionally, the model included three additional vaccination-related interventions that can be activated based on vaccine availability. Yao et al. [144] utilized DDQN to identify adaptive nonpharmaceutical interventions for controlling COVID-19 outbreaks and other respiratory infectious diseases. Using the required hospital beds to construct the state, they determined the threshold of available beds that would trigger stricter interventions.

**Table 1:** Summary of applications under the macrolevel research thrusts

Study	Year	Method$^{1}$	Epidemic model(s)$^{2}$ and data
General measures			
and strategies$^{3}$			
Das et al. [130]	2008	Simulation	N/A
Yaesoubi et al. [131]	2016	ADP	SIRD
Shi et al. [132]	2019	Simulation	N/A
Probert et al. [133]	2019	DQN (CNN)	SEIR
Liu et al. [134]	2023	QL (GNN)	SIS
COVID-19 control policies			
Uddin et al. [135]	2020	DQN	N/A
Arango et al. [136]	2020	DQN	SEIR
Miralles et al. [137]	2020	DQN	SEIR
Ohi et al. [119]	2020	DDQN (LSTM)	SEIR
Khadilkar et al. [138]	2020	DQN	SEIR
Kompella et al. [139]	2020	AC	SEIR
Padmanabhan et al. [140]	2021	QL	SEIR
Rathore et al. [141]	2021	Bayesian	SIR
Wan et al. [120]	2021	DQN, Monte Carlo, MARL	SEIR, SIR
Guo et al. [121]	2022	DQN (Transformer)	SEIAR, FluTE
Bushaj et al. [142]	2022	DQN	Covasim
Nguyen et al. [143]	2022	PPO	Agent-based simulation
Yao et al. [144]	2023	DDQN	SEIR, SIR
Mobility restriction policies			
Song et al. [145]	2020	DDPG (GNN)	SIHR, OD Matrix
Libin et al. [146]	2021	PPO, MARL	SEIR, Mobility Flux
Kwak et al. [147]	2021	D3QN	SIRD
Roy et al. [148]	2021	QL	SEIRD, Zone Mobility
Zong et al. [149]	2022	AC (RNN, Attention), MARL	SEAIRDL
Du et al. [150]	2023	Hierarchical PPO	Multilateral-impact-driven SEIR

Note:

1. “Method" refers to the learning algorithm (with approximator). The approximator in the bracket will be omitted if it is a standard setting of the RL algorithm (e.g., the standard approximator of DQN is DNN)

2. Under macrolevel research thrusts, RL agents generally interact with the epidemic model and use the model outputs as the state

3. COVID-19 studies are excluded from “general measures and strategies"

#### Mobility restriction policies

There has been a growing interest in studying mobility and travel policies during the pandemic. Libin et al. [146] investigated optimal policies for minimizing the number of susceptible individuals in different regions by integrating age groups within each region and mobility patterns between regions. They developed a country-wide MARL framework. A PPO algorithm was employed, with the available budget as a crucial control factor for both open and closed actions and for constructing the state variables. Their results suggested that the joint MARL approach consistently yielded lower costs. Kwak et al. [147] treated different countries as homogeneous entities and formulated the problem as a susceptible-infectious-recovered-dead (SIRD) model. By adopting diminishing rates of new infections, their algorithm recommended an earlier implementation of intensity strategies compared to the degrees of travel restrictions implemented by the government in each country.

In the context of urban mobility, Zong et al. [149] developed a sophisticated algorithm called the multi-agent recurrent attention actor-critic algorithm. Their case study focused on optimizing lockdown policies for different states in the US. Their algorithm interacted with a susceptible-exposed-asymptomatic-infected-recovered-death (SEAIRD) simulation model, incorporating heterogeneous locations such as schools, offices, and stores. The algorithm utilized a gated recurrent unit, setting it apart from and outperforming existing RL benchmarks such as [164]. Song et al. [145] aimed to identify mobility-control policies in Beijing that could simultaneously minimize the costs of infections and retain mobility. They achieved the objectives by developing a susceptible-infected-hospitalized-recovered (SIHR) model using real-world origin–destination (OD) data. The state, consisting of epidemic information and mobility demands, was fed into a GNN approximator within a DDPG framework. Their approach outperformed real-world expert policies in both early and late intervention scenarios by effectively addressing the life-or-economy dilemma, suppressing epidemics, and maintaining 76% of the mobility levels. Roy et al. [148] modulated zone mobility based on the healthcare system’s budget, estimated using local GDP. They employed queueing theory to analyze the hospitals in different boroughs of New York City, utilizing inter-zone mobility matrices. They proposed a QL algorithm to maximize mobility while considering the impact of high hospital occupancy. Through hierarchical RL [103], Du et al. [150] developed a multi-mode intervention strategy that integrates mobility constraints with medical resources and supplies as hierarchical actions to control the economic damage and contain the pandemic outbreaks. They also expanded a multilateral-impact-driven SEIR model to capture the impacts of different interventions. The optimal policies were assessed on two Chinese cities.

Table 1 summarizes the research studies under the macrolevel research thrusts. The applications of RL under the macrolevel research trust typically determine optimal healthcare policies, control critical epidemic conditions, and minimize overall costs within the constraints of available medical resources. The applications aim to strike a balance between hospital occupancy, infections, and economic impacts. RL functions by utilizing states from epidemic models and determining actions that encompass a range of epidemic interventions, such as social and travel restrictions or different levels of lockdown intensity. In these applications, the action space is usually discrete, leading to a more popular choice of DQN as the method. For infectious disease models, SEIR models [119, 120, 136–140, 146, 148, 160] are the most popular class that simulates the dynamic behaviors of epidemics.

One of the primary challenges in applying RL to healthcare policy is determining an effective reward function that accurately reflects real-world conditions. The impacts and rewards of interventions may be influenced by other factors, and validation can be expensive, with misspecification leading to incoherent learning. Real-time model updating with real-world data calibration or robustness optimization with uncertainties are potential solutions to this challenge. Furthermore, as more complex problems arise, more sophisticated RL algorithms can be deployed, such as those addressing large-scale multiple-wave epidemics, partially observable problems [131, 139, 141, 160], fine-grained policies, detecting super-spreaders, and immunity. However, practical implementations of DRL solutions in macrolevel applications remain rare in the real world. The rarity of real implementations is largely due to the high demands for transparency, trustworthiness, and regulatory compliance in these applications, prioritizing the interpretability of decision-making. Current studies only conduct sensitivity and statistical analyses of their policies. Designing interpretable RLs [165, 166] in low-dimensional representations that can address the dilemma of managing complex systems with strong interpretability remains a future direction.

### Mesolevel research thrusts

The mesolevel research thrusts cover operations such as distribution, resource allocation, organization design, logistics, and supply chain management within the healthcare services domain. This level of analysis serves as a bridge between the macrolevel and microlevel research thrusts. It operates within the framework of overall healthcare strategy but extends beyond the scope of a single healthcare institution [124]. The studies in this area can be classified into various domains, including humanitarian logistics, resource allocation during epidemics, and supply chain management in the healthcare industry.

#### Humanitarian logistics

Timely and effective decision-making is always crucial in providing relief after a disaster or mass casualty incident (MCI). Those situations are often challenged by partial observability and a high degree of uncertainty. RL-based approaches have been developed to aid humanitarian logistics, encompassing tasks such as distribution, rescue path searching, and transportation. These techniques facilitate humanitarian operations, enable rapid response, and enhance recovery efforts.

**Table 2:** Summary of applications under humanitarian logistics

Study	Year	Method	Problem class
Su et al. [47]	2011	QL	VRP
Nadi et al. [48]	2017	QL	VRP
Li et al. [167]	2020	Whittle’s restless bandit	Scheduling
Yang et al. [49]	2020	QL	VRP
Shin et al. [168]	2020	TD	Scheduling
Lee et al. [101]	2021	AC (RNN), imitation learning	Scheduling
Al-Abbasi et al. [65]	2021	DQN	Scheduling
Yu et al. [36]	2021	QL	Resource allocation
Fan et al. [169]	2022	DQN	Resource allocation
Van Steenbergen et al. [170]	2023	Value-based, policy-based RL	Resource allocation
Shen et al. [50]	2023	QL	VRP

Yu et al. [36] utilized QL for humanitarian distribution planning. Their objective was to minimize the delivery cost, the deprivation cost, and the terminal penalty cost. The local response center (modeled as an agent in their RL framework) decided how to allocate supplies to areas affected by disasters. Fan et al. [169] developed a DQN approach that takes into account the scarcity of emergency supplies. Through numerical experiments, they demonstrated the effectiveness of RL in terms of computational time and objective values, particularly in tackling problems with high-dimensional spaces. In another study, Van Steenbergen et al. [170] introduced Unmanned Aerial Vehicles (UAVs) to humanitarian complement trucks and optimized multi-vehicle, multi-trip, split-delivery routes under travel time uncertainty. By evaluating both value function approximation and policy function approximation, they validated that dynamic methods and UAV deployment significantly enhance operational performance and robustness, particularly in reaching remote locations.

In a problem of rescue path selection, Su et al. [47] utilized a rectangular grid to represent the affected area. They implemented an RL framework where the rescue team was represented as an RL agent. The team aimed to find the most efficient path connecting two points, and a mechanism for escaping cyclic paths was incorporated into the design. Nadi et al. [48] improved a MARL framework by incorporating relief assessment and emergency response teams in an online setting. The relief assessment teams utilized a prediction model to estimate the demands in affected areas. The response team then solved a vehicle routing problem (VRP), considering the requests’ priorities and both teams’ capacity and time window constraints. Shen et al. [50] modeled an aviation emergency rescue problem with a stochastic game process and employed MARL to determine task acceptance/rejection decisions at different locations. Yang et al. [49] proposed a MARL approach, coined ResQ, for disaster response. This framework utilized Twitter data related to the specific disaster to map the geo-locations of volunteers and victims. The states in the framework included the volunteers’ spatial and temporal information, which served as inputs for the heuristic allocation strategy. The reward function, controlled by the total distances from agents to victims, was optimized using QL in a POMDP setting.

Another aspect of humanitarian logistics is the transportation of patients to healthcare facilities after MCI. Effective triage and prioritization are crucial to saving lives, but it is a computationally demanding task. Li et al. [167] studied Whittle’s restless bandits approach to learning triage and other relevant decisions over a finite but uncertain time horizon. The number of bandits would, therefore, change over time. Because of the stochastic nature of this problem, the authors proposed novel lagrangian relaxation methods to decompose the original problem, which have gained significantly higher performance. Lee et al. [101] developed a MARL framework powered by imitation learning to address the problem. Their goal was to maximize the number of survivors in MCIs by optimizing the decisions related to patient admissions to emergency departments (EDs) and diversion of patients. Unlike previous studies focusing on individual patient assignment in outpatient care, this problem involved coordinating multiple homogeneous cooperative EDs (represented as agents in the MARL framework). Each agent only had partial information, such as the current patient arrivals, patient conditions, and its individual available beds (in the ED). Positive rewards were accumulated based on the survival probability of admitted patients, which was determined by their health conditions. An AC approach was used for a multi-agent setting, and the historical actions and realizations were inputted into an RNN to determine current actions. A policy gradient algorithm was implemented based on a generalized advantage estimator (GAE) [78]. Behavioral cloning was employed as a conceptual optimization method using integer programming to pre-train the neural networks. This imitation learning technique helps reduce computational time and yields a high-quality policy. Additionally, a meta-algorithm, subspace partitioning, was utilized as another optimizing approach, as discussed by Shin and Lee [168]. Another study by Al-Abbasi et al. [65] also considered a patient transportation problem across heterogeneous medical facilities, where they used DQN to train their model.

As presented in Table 2, the earlier applications in humanitarian logistics utilized QL for solving classes of VRP. Subsequent studies incorporated DNN to construct DRL frameworks. Neural networks’ strong predictive capabilities enable the development of more sophisticated models with increased performance, for example, by integrating multiple agents and behavioral models to guide action selection. The combination of MARL under the framework of POMDP has shown significant potential in disaster relief [48–50, 101]. Once these complex models are well-trained, RL can provide rapid responses in a short time. Moreover, leveraging imitation learning from an expert policy is anticipated to improve training efficiency. Learning from experts can also aid in extracting domain knowledge, leading to improved interpretability. As such, DRL offers an efficient solution for tackling complex humanitarian logistics challenges.

#### Resource allocation in epidemics

While we primarily discussed research on epidemics within the macrolevel research thrusts in Sect. 4.1, we also acknowledge a few RL applications for epidemics, specifically focusing on disaster response [114], as studies falling within the mesolevel research thrusts. In contrast to the epidemic control and healthcare strategies under the macrolevel research thrusts, the topics discussed in this subsection primarily focus on addressing logistics and resource allocation challenges during pandemics.

**Table 3:** Summary of applications under resource allocation in epidemics

Study	Year	Method	Application
Wei et al. [171]	2021	QL, AC	Vaccine allocation
Hao et al. [172]	2021	QL (CNN), DQN	Vaccine allocation
Tan et al. [173]	2021	QL, DQN	Vaccine allocation
Bednarski et al. [174]	2021	QL, Value-based RL	Ventilator redistribution
Bastani et al. [88]	2021	MAB (Bayes), lasso, gradient boost	Test kits allocation
Gonsalves et al. [89]	2021	MAB (ICAR)	Testing priority
Hao et al. [175]	2022	PPO (GNN)	Vaccine allocation
Shuvo et al. [74]	2022	A2C, Pareto optimality	Hospital expansions
Xia et al. [176]	2022	DQN	Vaccine and test kits allocation
Rey et al. [90]	2023	MAB	Vaccine allocation
Thul et al. [177]	2023	Stochastic optimization	Vaccine and test kits allocation
Zeng et al. [178]	2023	DQN	Medical supplies allocation
Zhang et al. [179]	2023	DQN	Vaccine allocation

During a pandemic, two crucially scarce medical resources are test kits and vaccines. Focusing on test kit allocations, Bastani et al. [88] introduced “Eva" as a solution to allocate limited test kits to different groups of arrivals at Greek borders. The problem was initially formulated using MAB, where the prevalence of COVID-19 was estimated through an empirical Bayes approach. Subsequently, certainty-equivalent updates and an optimistic Gittins index were utilized to guide allocation decisions. In the approximation phase, Lasso feature selection [180] was employed to handle the high dimensionality. The “Eva" RL system was evaluated using counterfactual analysis based on inverse propensity weight scoring [181]. Additionally, the authors compared the predictive power of epidemiological metrics in gradient boosting [182] by incorporating different combinations of features and conducting comprehensive estimations and validations. Gonsalves et al. [89] introduced an intrinsic conditional autoregressive prior distribution and a hierarchical Bayesian strategy. They utilized mobility data from UberMedia to identify potential testing locations.

In the context of vaccine allocations during pandemics, RL agents consider information about various population groups categorized by geographical locations and ages. They utilize such information to determine the allocation of vaccines, considering resource scarcity. The objectives of these RL agents are mainly to minimize the number of infectious cases, maximize the number of critical patients treated, and optimize the economic impacts [171]. Hao et al. [172] introduced a hierarchical RL model that addresses the simultaneous allocation of vaccines and beds. To mitigate computational costs, they implemented various ranking strategies to filter regions based on specific pandemic thresholds. Other studies have also explored different approaches to vaccine allocation. Tan et al. [173] employed a random forest algorithm [183] with real-world data to predict future infections before making vaccine allocation decisions. Hao et al. [175] went beyond using a simple approach for simulation and relying solely on a black box approach. They instead incorporated expert solutions to enhance the performance of their RL model. Additionally, they conducted a sensitivity analysis to improve the model’s explainability. More recent studies have focused on developing MARL methods for vaccine allocation. Rey et al. [90] employed a budget-sharing mechanism to improve performance with Thompson Sampling [184].

By integrating a SIR model and a stochastic block model network, Xia et al. [176] proposed a degree-based testing and vaccination model. They employed both Pontryagin’s maximum principle [185] and DQN to optimize the control strategies. Zeng et al. [178] enhanced the medical supplies dispatching process by incorporating additional states such as “asymptomatic", “hospitalized", and “deceased" into their SEIR model. They utilized a DQN structure to optimize the dispatch decisions. Thul et al. [177] introduced a stochastic optimization approach for vaccine allocation. They considered a collaborative environment where a vaccination agent and a learning agent interactively determine the allocation of stockpiles of vaccines and tests to a set of zones. The learning agent makes decisions regarding the allocation of test kits and utilizes the belief state to inform the vaccination agent. The authors proposed an optimal policy using a parameterized direct lookahead approximation based on Bayesian optimization. Their approach demonstrated superior performance compared to value function approximations, and greater scalability and robustness in both COVID-19 and nursing home contexts.

Regarding the allocation of other medical resources during pandemics, Bednarski et al. [174] and Zhang et al. [179] explored the use of value-based RL for the redistribution of ventilators to alleviate shortages and reduce costs. They utilized LSTM models with logistics downtime to infer real-time demands across different states. Shuvo et al. [73, 186] optimized the decision-making process for hospital expansions by considering socioeconomic indicators and current capacities, applicable to both pandemic and non-pandemic scenarios. Their studies aimed to minimize costs associated with capacity expansion while simultaneously reducing the occurrence of denial of service (DoS) situations. To forecast hospital occupancy, various regression models were employed, and the most suitable model was selected for downstream planning.

Table 3 summarizes the applications that address decision-making problems related to resource allocation in epidemics, which mainly involve allocating medical resources based on spatial and temporal states. In addition to the popular value-based and policy-based RL methods, we also highlight the widespread use of MAB algorithms in allocating test kits and vaccines during epidemics due to their scalability. Furthermore, the interpretability of MAB algorithms is enhanced through the use of cooperated linear models [88, 89]. In some cases [176, 177], robust optimization methods have demonstrated superior performance compared to basic RL methods. This has inspired the exploration of combining robust optimization and RL, with the former pre-training and restricting the action space [187] for RL to achieve higher solving efficiency and rewards, especially in large-scale problems.

#### Healthcare supply chain management

While RL for healthcare supply chain management is a relatively nascent area, recent studies have begun to delve into various applications to aid decision-making in healthcare supply chain operations.

**Table 4:** Summary of applications under healthcare supply chain management

Study	Year	Method	Application
Wu et al. [188]	2020	Policy gradient (LSTM)	Mask production scheduling
Abu Zwaida et al. [189]	2021	DQN (CNN)	Replenishment of medicine
Asadi et al. [190]	2022	Value iteration	Routing for delivery by drones
Ahmadi et al. [191]	2022	DQN	Inventory control of perishable medicine
Abouee-Mehrizi et al. [45]	2023	ADP	Inventory control of platelet
Zhang et al. [105]	2023	Hierarchical AC	Ventilator production scheduling
Seranilla and Löhndorf [192]	2023	ADP	Facility location for vaccine distribution
Wang et al. [66]	2023	DQN	Medical facility location routing
Tseng et al. [75]	2023	AC	Inventory control of regenerative medicine
Vanvuchelen et al. [193]	2023	PPO	Inventory control of malaria medicine

**Table 5:** Summary of applications in EMSs

Study	Year	Method	Application
Maxwell et al. [194]	2010	ADP	Ambulance redeployment
Schmid [195]	2012	ADP	Ambulance dispatch and relocation
Maxwell et al. [196]	2013	ADP	Ambulance redeployment
Nasrollahzadeh et al. [197]	2018	ADP	Ambulance dispatch and relocation
Allen et al. [198]	2021	Simulation	Ambulance location problem
Benedetti et al. [199]	2021	DQN	Traffic light timing
Yu et al. [200]	2021	ADP	Ambulance dispatch
Gao et al. [201]	2023	ADP (DNN)	Ambulance dispatch and relocation
Su et al. [102]	2023	A2C (LSTM)	Traffic signal control

One such paper is Wu et al. [188], which addressed a production scheduling problem for medical products. Their proposed algorithm utilizes LSTM as an approximator and policy gradient to schedule the production of medical masks. A study conducted by Zhang et al. [105] introduced a system of flexible production scheduling specifically designed for ventilators. Their proposed framework employed a hierarchical RL approach, utilizing heterogeneous digital twin agents to enhance collaboration efficiency between humans and machines. It is worth noting that this line of research often relies on simulation results, which can involve more realistic scenarios. Asadi et al. [190] studied the supply of critical medical needs, focusing on optimizing routing and delivery. They utilized RL to tackle a medical drone delivery problem, where the RL agent considered battery levels and demands to optimize recharging actions. The objective was to maximize the expected satisfied demand. Seranilla and Löhndorf [192] considered the possibility of facility failures due to natural disasters. They utilized shadow price approximation for a multistage stochastic vaccine facility location problem. Their proposed ADP approach successfully reduced operational and transportation costs by 30%. Wang et al. [66] addressed location routing problems for emergency facilities with a two-stage DRL approach.

Inventory management of medical products is another area of interest. Using DQN, Ahmadi et al. [191] examined inventory decisions for perishable pharmaceutical products. Similarly, Abu et al. [189] investigated a standard replenishment problem in a medical supply chain, where a DQN agent determines whether to refill or not to minimize refilling costs, storage costs, and shortage costs. Tseng et al. [75] utilized AC to facilitate dynamic capacity planning of decentralized regenerative medicine. Van Vuchelen et al. [193] optimized health facility stock management through PPO. Their derived transshipment policies enhanced service level equity, particularly in resource-constrained environments, and were robust given demand seasonality. Recently, Abouee-Mehrizi et al. [45] studied a stochastic perishable inventory control problem for blood platelets, where the shelf-life of delivered units is uncertain and potentially depends on the order size. Their ADP-based blood platelets ordering policy, which approximates a non-convex value function using basis functions and simulation-based policy iteration, significantly outperforms historical hospital performance and other benchmarks in a case study using real data from Canadian hospitals. With comprehensive numerical experiments, their study has made valuable contributions to platelet inventory management under uncertainty.

The applications of RL for healthcare supply chain management are summarized in Table 4. The table indicates that this field is an emerging area and presents diverse applications. These applications span various areas, including production, routing, and inventory management. Moreover, they are solved through a variety of RL approaches. From our review, studies in healthcare supply chain management have utilized conventional value-based and policy-based RL methods to optimize medicine replenishment and transportation decisions. Recent research has also explored adopting a hierarchical framework [105] and leveraged QL as an adaptive heuristic approach to accelerate the convergence of medical supplies scheduling [118]. However, there is still significant potential for further exploration and applying more efficient methods. Such advancements are expected to yield substantial benefits for the healthcare service industry.

### Microlevel research thrusts

The most prominent level of HOM research is the microlevel research thrusts, as suggested by a number of studies [124, 127, 128]. At this level, HOM research problems are analyzed at the individual patient level within a single healthcare institution. Most of the approaches to tackling the problems take into account the specific needs of each patient and provide more detailed plans for healthcare service delivery. The studies under the microlevel research thrusts typically investigate four main categories of healthcare services, including emergency medical services (EMSs), outpatient care, inpatient care, and residential care.

#### Emergency medical services

Managing a fleet of emergency ambulances efficiently can be difficult due to their limited availability and the unpredictable distribution of emergency calls regarding location and time. In the past, researchers mainly focused on static policies for ambulance dispatch. With technological advancements, there is a growing interest in studying dynamic vehicle operations. One popular method in the field is the development of ADP approaches using basis functions for approximation (as summarized in Table 5). Several formulations have been proposed to address problems in different scenarios.

In a pioneering study by Maxwell et al. [194], an ADP-based model and a greedy heuristic for dispatch assignments were proposed for ambulance redeployment. The paper also considered call center management, where a request is lost if all line pickers are busy. The objective was to simultaneously minimize the total number of missed calls, total response time, and relocation costs. The authors utilized direct search [196] to fine-tune their ADP policies. Subsequent works aimed to incorporate ADP into both relocation and dispatch decisions. Schmid et al. [195] proposed an ADP algorithm that dynamically relocates and dispatches vehicles, aiming to minimize the total response time of all requests under stochastic travel time and changing request volumes. Nasrollahzadeh et al. [197] studied a similar problem and applied real data. Another study utilized a first-order stochastic dominance method to enhance the robustness of solutions [200]. One of the challenges in this research area is the development of a comprehensive environment to simulate arrivals, relocation outcomes, and dispatch processes, which can be time-consuming. To address the challenges, Allen et al. [198] developed a complete gym-compatible environment for this problem. This environment involves multiple vehicles, dispatch centers, and patients, enabling the simulation of the entire ambulance dispatch process. In recent work by Gao et al. [201], ambulances were effectively coordinated with UAVs using DNN-based policy iteration. The objective was to minimize EMS response times for better patient health outcomes. The action space was event-based, depending on the state constructed from queueing, temporal, and geographic properties. The authors particularly emphasized their optimal policies when facing surge demands.

Instead of focusing on the operations of ambulance fleets, Benedetti et al. [199] studied the application of DQN to a traffic management problem with emergency vehicles. Here, the DQN agent learns the status of the lane and controls traffic lights to reduce the waiting time for emergency vehicles. Su et al. [102] designed a MARL framework that combines emergency vehicle routing with traffic signal control and minimizes travel times of both emergency vehicles and other vehicles by measuring their introduced lane pressure.

Henderson et al. [202] highlighted the challenges faced by the EMS systems, including issues like traffic congestion, heterogeneous vehicles, and the growing volume of emergency calls. Their review provided an overview of widely utilized methods to address these challenges, including real-time optimization, offline optimization, stochastic DP, and ADP.

#### Outpatient care

Outpatient care, also known as ambulatory care [128, 203], refers to a range of medical services provided without requiring hospital admission. In an RL framework, one notable characteristic of outpatient care is that an episode representing patient care generally involves one or multiple visits to healthcare facilities within the same day. Typical examples of RL applications for outpatient care include patients visiting EDs, laboratories, surgical centers, or diagnostic centers. In these settings, healthcare organizations aim to satisfy the demands for services. Given the capacity limitations and resource constraints in outpatient departments, optimization is needed. In recent years, researchers have developed RL and ADP approaches to address the challenges in outpatient care. These approaches have been applied in a range of applications to optimize resource allocation and improve the efficiency of outpatient services. The main challenge revolves around patient scheduling for outpatient resources or facilities, with the underlying objective of selecting or prioritizing patients effectively.

Patrick et al. [204] were among the first to employ ADP for cost-effectively achieving wait-time targets in patient scheduling for computerized tomography (CT) scanners. Their approach involved making decisions on available appointment slots to assign to waiting demand units, considering stochastic patient arrivals. Huang et al. [205] extended the research by applying QL to a business process management model for resource allocation, using radiology CT-scan examination procedures as a case study. Lee et al. [210] focused on detecting hepatocellular carcinoma within the constraints of screening capacity. They employed greedy, interval estimation, and Boltzmann exploration techniques to maximize the number of detected cancers and generate risky ranks for patients. They further improved their methodology by incorporating an MAB framework [91]. Each bandit represented a POMDP, and one patient was selected for screening in each decision epoch based on health state estimations. Transition matrices for screened and unscreened patients were constructed separately within the clinical system. The proposed optimal policy resulted in detecting 22% more early-stage cancer cases and suggested outpatient decision-making with a truncated planning horizon. Lee et al. [211] applied DQN to make assignments of patients to different medical resources, including X-ray or CT scanners and consultants. The state information included patients’ demands and acuity levels. By adapting DQN, their approach prioritized risky patients and minimized waiting times, outperforming conventional scheduling rules. Recently, Zhalechian et al. [92] made contributions to research in the application of online learning and for healthcare resource allocation. They introduced a novel and generic framework that synergizes contextual learning with online allocation mechanisms to enable personalized decision-making under uncertainty. Besides the exploration-exploitation trade-off, their proposed algorithms address critical challenges, such as adversarial customer arrivals, stochastic rewards and resource consumption, and delayed feedback, with performance guarantees. An online advance scheduling algorithm, which incorporates multiday booking and no-show behavior, demonstrates strong performance theoretically and empirically using real data from their collaborating health organization.

Astaraky et al. [209] presented a surgical scheduling problem, taking into account the availability of operating rooms and recovery beds. Their objective was to minimize the complexity and cost of bookings by determining the number of advanced days for patients to book. They used a least-square iteration method to fine-tune the approximation parameters for state vectors, which include the master schedule, booking slate, hospital census, and waiting demand. This approach was compared to a FIFO scheduling policy, and their proposed ADP policy consistently outperformed the FIFO policy in both high and low system capacity scenarios. Zhang et al. [213] designed a recursive least-squares TD algorithm to balance waiting times and the over-utilization of surgical resources. Decisions were made on a weekly basis to select which patients would be treated. The MDP state was defined by patients’ groups, required specialties, maximum recommended waiting times, and the number of associated patients. The objective was to minimize surgery costs and delays. They also incorporated structural analysis into the ADP framework to improve efficiency by generating a feasible action subspace. In more recent studies, Xu et al. [44] addressed the backlog of elective surgeries caused by disruptions during the pandemic. They applied a model-based piecewise decaying $\epsilon$-greedy RL approach with an auxiliary system [216] to minimize the time required to clear the surgical backlog and restore surgical activity. A queueing network system consisting of a backlog queue and a newly arrived queue was formulated as a countable-state MDP. Dynamic patient scheduling for these two queues was implemented based on patients’ clinical urgency. In the context of the pandemic, D’Aeth et al. [215, 217] developed an optimal nationwide prioritization scheme. They modeled each individual as a DP considering each patient’s health status and aggregated all individuals as a grouped weakly coupled DP with global constraints (e.g., hospital beds, doctors, and nurses). Treatment options, such as prioritizing specific disease patients, were determined for each individual to maximize the overall years of life gained nationwide.

**Table 6:** Summary of applications in outpatient care

Study	Year	Method	Application
Patrick et al. [204]	2008	ADP	Diagnostic resource management
Huang et al. [205]	2011	QL	Diagnostic resource management
Lin et al. [206]	2011	ADP	Outpatient appointment scheduling
Schuetz et al. [207]	2012	ADP	Capacity allocation
Feldman et al. [208]	2014	ADP	Outpatient appointment scheduling
Astaraky et al. [209]	2015	ADP	Surgery scheduling
Lee et al. [210]	2015	Boltzmann exploration	Diagnostic resource management
Lee et al. [91]	2019	MAB	Diagnostic resource management
Lee et al. [211]	2020	DQN	Diagnostic resource management
Diamant et al. [212]	2021	ADP	Outpatient appointment scheduling
Zhang et al. [213]	2021	TD	Surgery scheduling
Zhalechian et al. [92]	2022	MAB	Diagnostic resource management
Agrawal et al. [214]	2023	ADP	Surgery scheduling
Xu et al. [44]	2023	Model-based RL	Surgery scheduling
D’Aeth et al. [215]	2023	DP (fluid approximation)	Care prioritization

In appointment scheduling, Lin et al. [206] utilized aggregation and Monte Carlo simulation to determine slot assignments for call-in patients with different no-show rates. Feldman et al. [208] investigated preference-based healthcare plans and customized appointments. They moved from a static model to a dynamic model that considers patients’ no-show behavior and proposed a heuristic solution. Diamant et al. [212] formulated a multistage patient scheduling problem as a rolling-horizon MDP. Their approach described different types of patients undergoing specific care plans consisting of a series of assessments or treatments. The state provided patient-centered care plans, including no-shows and patients who rescheduled, to maximize the number of patients who could successfully complete all stages of treatments. Patients’ arrivals, referrals, and ineligibility rates were modeled using statistical distributions, and dual variable aggregation helped efficiently solve the large-scale linear programming model. This work is built upon earlier research on variable aggregation [218]. Schuetz et al. [207] considered the costs of rejecting a request, no-shows, and overtime in appointment scheduling. They used ADP to decide whether to accept or reject a new request from a class-type combination (patient and examination classes). Agrawal et al. [214] proposed an ADP approach that takes patients’ requests of “dedicated," “flexible," and “urgent" (which must be met on the same day) to determine appointment decisions. Their objective was to maximize revenue and minimize physician overtime and idle time while satisfying as much demand as possible.

Table 6 provides a summary of research studies in outpatient care discussed in this section. Among these applications, ADP is one of the most popular methods for optimizing outpatient service delivery. This model-based approach has been simulated and validated in clinics and hospitals of different scales [204, 209] and has consistently outperformed heuristic algorithms regarding total costs, while consuming less computing time than DP. Different RL methods have also been compared in the existing studies. For example, in Diamant et al. [212], ADP outperformed A2C and greedy algorithms regarding rewards for the featured patient group. These findings suggest that RL approaches require more research efforts to adapt to domain-specific settings in outpatient care [219]. Integrating model-based [44] and dimensionality reduction methods [220] is expected to solve more specific and complex problems. An interesting and important future direction is accommodating dynamic changes in factors such as hospital capacities, patient preferences, and doctor preferences to enable real-time operations.

#### Inpatient care

Inpatient care primarily encompasses the management of patient flow and related HOM that take place in inpatient wards. This includes admitting and discharging patients, transferring patients between specialty wards, and estimating patient LOS. In recent years, researchers [221, 222] have conducted extensive reviews of the latest modeling and analytical techniques for inpatient management. Our current review also finds that solutions utilizing ADP and RL have demonstrated substantial potential in enhancing inpatient care.

**Table 7:** Summary of applications in inpatient care

Study	Year	Method	Application
Samiedaluie et al. [223]	2017	ADP (queue theory)	Inpatient flow management
Prasad et al. [224]	2017	QL	Weaning of mechanical ventilation in ICU
Dai et al. [30]	2019	ADP (fluid control, single-pool approximation)	Inpatient flow management
Braverman et al. [225]	2020	ADP (Taylor expansion)	Inpatient flow management
Shuvo et al. [186]	2020	A2C	Hospital capacity expansion
Shuvo et al. [73]	2021	A2C, decision tree	Hospital capacity expansion
Kabir et al. [226]	2021	A2C (LSTM)	Hospital capacity expansion
Heydar et al. [227]	2021	ADP	Inpatient flow management
Liu et al. [228]	2021	MAB	Inpatient flow management
Lazebnik [229]	2023	Policy-based RL	Hospital staff scheduling
Zhalechian et al. [122]	2023	MAB	Inpatient flow management

Samiedaluie et al. [223] developed a queue theory-based ADP approach to manage stroke patients in the neurology ward effectively. The state information involved the number of patients with different severity levels and occupied beds. The objective was to minimize waiting and transferring costs, taking into account the quality of life determined by discharge destinations. The authors also incorporated a priority cutoff policy during the experimental phase to facilitate the implementation of the ADP solution. In a similar problem, Dai et al. [30] modeled inpatient operations as a multi-pool queueing system and combined fluid control with single-pool approximation in their ADP approach. Their aim was to minimize the costs associated with the inpatient overflow policy. To tackle the computational challenge, they utilized the basis function for the midnight time epoch to guide the basis functions for other time epochs, when approximating value functions using admission and discharge information. Heydar et al. [227] formulated the patient-to-bed problem to determine the next-best decision when the most appropriate ward was unavailable, considering random arrivals and inpatient LOS. They employed linear approximations supported by features related to patients and wards in their ADP approach, while using phase-type distributions to model the LOS. In general, ADP policies demonstrated a significant reduction in boarding time from ED and effectively controlled total costs compared to popular existing strategies. Braverman et al. [225] created an ADP solution based on Stein’s method [230] and implemented it in an inpatient overflow experiment (presented in Dai et al. [30]). The suboptimality of the solution was established conceptually using the Taylor equation. In another study, Liu et al. [228] assessed their constrained linear bandits approach for managing inpatient overflow considering fairness. Following their prior work on the application of MAB for outpatient [92], Zhalechian et al. [122] proposed a data-driven algorithm for a hospitals’ admission control problem where the patients’ lengths of stay are uncertain, given limited reusable inpatient beds. Their data-driven admission control algorithm is designed to adaptively learn the readmission risk of different patients through batch learning with delayed feedback and choose the best care unit placement for a patient based on the observed information and the occupancy level of the care units. The performance measure of this online algorithm is Bayesian regret, and the Bayesian regret bound is also proved. With experiments on data from a healthcare system, their results show an improved performance compared to traditional admission control methods. Their paper highlights the potential benefits of using data-driven approaches in healthcare and suggests that this insightful approach can be further improved with enhanced data quality and volume and algorithms.

In ICU management, Prasad et al. [224] proposed a QL approach to optimize the weaning process of mechanical ventilation. They considered a 32-dimensional representation of the patient state incorporating as many useful and easily accessible features as possible. Actions to determine whether to have the patient off or on the ventilator and the level of sedation to be administered over the next 10-minute interval are determined at each stage. This innovative approach was tested on real patient data and has shown promising results in minimizing reintubation rates and regulating physiological stability.

Shuvo et al. [186] conducted a study on determining the optimal timing for increasing the number of beds in hospitals for upgrade. They considered the current capacity and the growth of the patient population, aiming to minimize costs associated with untreated patients and the maintenance of additional beds. With a comparison with myopic policies, their proposed A2C approach yielded the lowest costs. Subsequently, they extended their research by incorporating multiple hospitals in different geographic regions and including age information in the state space [73]. By utilizing real-world data, they were able to improve the effectiveness of their proposed approach using decision tree regression and predict population growth using models [226].

RL has also been applied for staff scheduling problems for inpatient operations. Lazebnik [229] enhanced staff schedules by employing agent-based simulation and policy gradient approaches with the *rmsprop* algorithm [231]. This approach demonstrated improved resilience to anomalies. The study also revealed a second-order polynomial relationship between successful treatment and budget.

Table 7 provides an overview of the applications of RL in inpatient care. The most popular approaches include ADP and A2C, which are well-suited for capturing the dynamic nature of inpatient operations, such as modeling inpatient flow. RL models often utilize queueing models to estimate queue lengths and waiting times, which are essential for making informed decisions regarding inpatient admission and discharge. As we have reviewed in this subsection, the applications of RL for inpatient care have shown promise in recent studies. The main objectives of these studies were to minimize patient boarding, reduce the time patients spend in the hospital, and avoid associated penalties while maintaining the quality of care and improving inpatient outcomes. Accurate estimation of patient arrivals and demands is crucial, and various effective forecasting regressions and statistical inferences can be utilized. Downstream optimization methods would also need to be designed so that estimation errors are considered. However, selecting the most appropriate basis function for ADP (or the approximator for RL) remains a challenge, as it depends on the characteristics of the inpatient operations. Therefore, conducting experimental trials and comparisons is necessary to enhance the RL approaches’ effectiveness. Future research could combine inpatient, outpatient, and other hospital processes into a more complex interactive system to guide better decision-making. Additionally, incorporating human behaviors and preferences into modeling inpatient operations, as done in outpatient care studies, could be valuable.

#### Residential care

Residential care involves providing personalized healthcare services to patients within the comfort of their own homes [232]. This approach enables individuals to maintain their independence and enhance their quality of life [233].

Cire and Diamant [232] developed an ADP approach to optimize the assignment of health practitioners (HPs) to patients. They compared four policies and found that the models based on fluid approximations [234] outperformed those that utilized heuristics. Their methodology demonstrated superior performance compared to commonly used constrained versions of VRP when accounting for future uncertainty. Their framework involved deciding whether to accept or reject a patient referral and assigning an HP to the patient if the decision is accepted while accounting for resources, care continuity, and time windows. The policy for arranging HPs working in a small set of adjacent regions aimed to maximize the expected long-term cost savings while minimizing the number of rejected referrals. In another study, Salehi et al. [235] combined RL with a functional resonance analysis method (FRAM) to explore complex operations. They deployed an RL agent to examine 38 functions (such as “access the patient," “go home without services," “invite a caregiver," etc.) and incentivized it to select the optimal functional routes based on the patient’s health improvement.

In recent years, the Internet of Medical Things (IoMT) has been increasingly utilized in residential care [236, 237]. IoMT refers to a network that integrates medical devices, sensors, learning algorithms, and mobile health technologies. Through IoMT, healthcare institutes can collect real-time health information, provide remote services, and provide personalized interfaces [238, 239]. To improve the quality of service (QoS) of IoMT facilities, a number of RL-based technologies, including blockchain [240], cloud systems [241], and fog computing [242], have been developed in the research community of telecommunications. RL-based wearable devices can also provide customized support for patients’ rehabilitation [233, 243]. By reminding or alerting patients in their daily lives, RL assistance is expected to guarantee high-quality residential care for impaired patients and reduce the burden on their caregivers [244]. In the OR community, queueing theory has been utilized to optimize the matching process between patients and medical resources, such as specialists, in cloud healthcare systems. The objective was to minimize the total medical costs [245]. Tiwari et al. [246] utilized a combination of MARL and Federated Learning [247] to minimize the latency of an IoMT system. Seid et al. [248] used a similar learning method to minimize the energy consumption of a drone-enabled healthcare system. Chen et al. [249] optimized task offloading in wireless body area networks using a DDPG-based strategy and mobile edge computing servers for IoMT.

Based on our review, we observe the number of studies with the deployment of model-based ADP and MARP techniques in residential care [232, 245, 246, 248]. These studies are also of interest to other disciplines, such as telecommunications and electronics. The rapidly growing and multi-disciplinary field of IoMT is expected to revolutionize residential care by facilitating remote patient monitoring, personalized medical recommendations, and the applications of OR for HOM.

### Medical treatments

It is important to distinguish HOM from some other similar areas where RL has also been widely used in recent years. As stated in Sect. 3, our review analyzes existing HOM research as described in the healthcare ecosystem map, where non-HOM research studies focusing on medical imaging and medical robotics for medical treatments are excluded. These excluded studies often involve advanced computer vision and robotics techniques that may differ significantly from the use of ADP and RL in HOM. For more comprehensive reviews focusing on medical treatments, we refer the reader to [4, 250–252].

Another area that is related to, yet different from HOM, is dynamic treatment regimes, which pertain to detailed treatment strategies for patients in hospitals, healthcare facilities, and patient homes [253]. RL-based clinical decision-making has proven beneficial in assisting medical staff with tasks such as determining dosing regimes for chemotherapy in clinical trials [254], split liver transplantation [255], treating Parkinson’s disease [243, 256], diagnosing skin cancer [257], and managing glycemic control in Type 2 diabetes [258]. Fatemi et al. [259] used DQN to identify medical dead-ends of patients’ sequential treatments and avoid risky states for treatment security. Bennett et al. [260] demonstrated the benefits of their proximal RL approach in a POMDP setting for sepsis management [261].

Under the umbrella of medical decision-making, dynamic treatment regimes are having more and more RL applications. This section is only intended to exemplify a few insightful studies, as there are still numerous explorations and positive outcomes coming in this field. For more comprehensive reviews on this topic, we refer the reader to [5, 39, 262].

## Trends and directions

Through our scoping review, we have collected statistics to visualize the overall trend of RL applications in HOM. In this section, a critical discussion of the current development that covers the performance of various RL methods for corresponding HOM problems is presented. Additionally, we address the challenges faced in this field and discuss insightful future directions for RL applications in HOM.

**Fig. 1:**
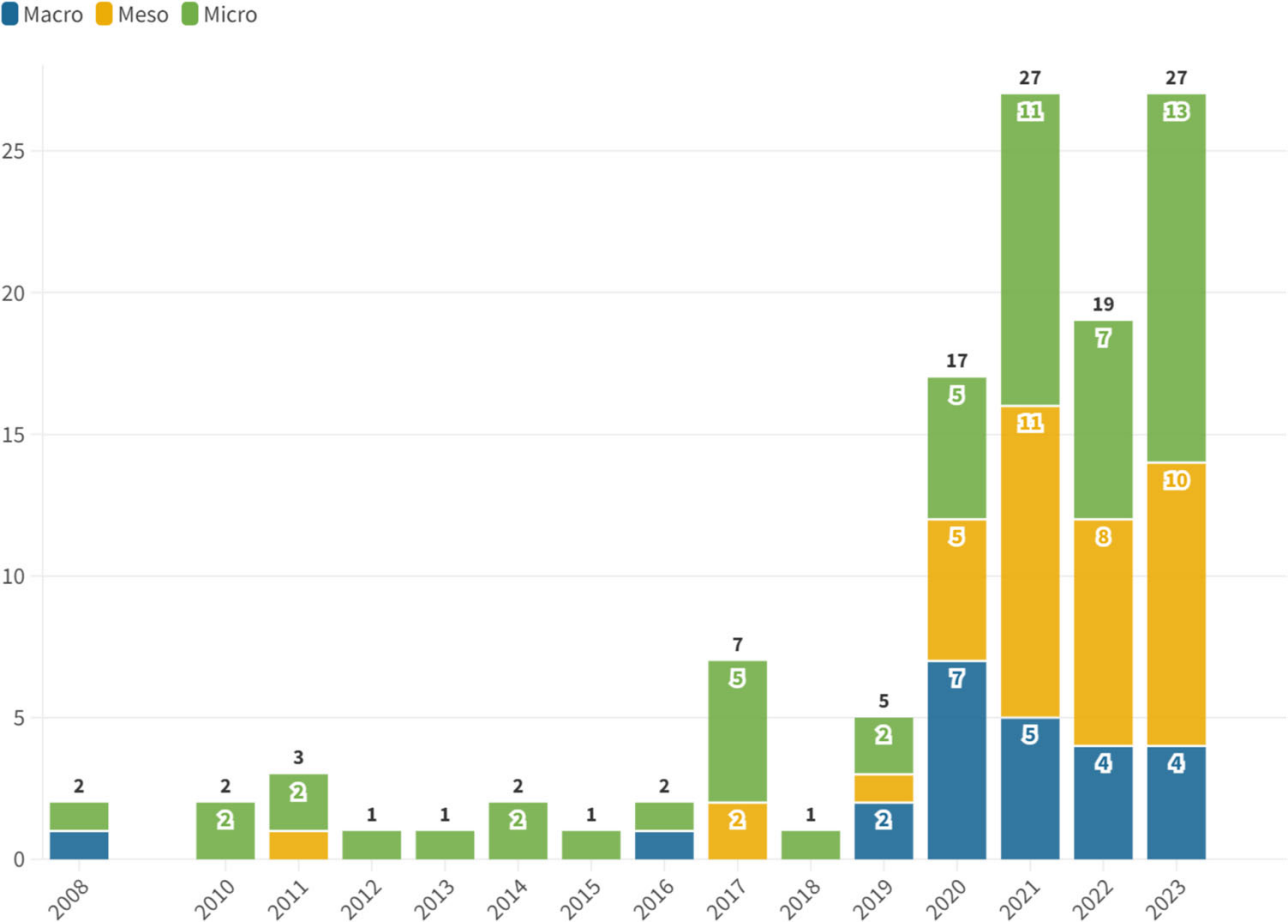
Number of publications related to RL for HOM by year

### Statistics

Fig. 1 presents the trend of the number of publications related to RL applications in HOM. The earliest related studies date back to 2008. These early studies initially utilized methods from optimal control and DP, which align with the RL paradigm. The number of publications remained steady at around one to two studies per year until 2016 when RL algorithms demonstrated mastery in the game of Go [263]. In 2017, there was a peak in the use of RL in mesolevel and microlevel research thrusts. Since then, there has been exponential growth in publications, which has continued until the time of this review. This suggests that RL is becoming increasingly established and effective in solving HOM problems.

In terms of the number of publications at each division level, we reviewed 24, 38, and 55 papers under macrolevel, mesolevel, and microlevel research thrusts, respectively. The rapid growth of publications under the marcolevel research thrusts started in 2019, which could be attributed to the COVID-19 pandemic. Researchers actively explored the potential of RL in optimizing macrolevel policies associated with healthcare to manage this pandemic better. Similarly, mesolevel applications, which are mostly related to resource allocation and supply chain management, experienced a significant increase after 2019. It has been proven that RL can powerfully assist decision-making during pandemics in practice [88].

**Fig. 2:**
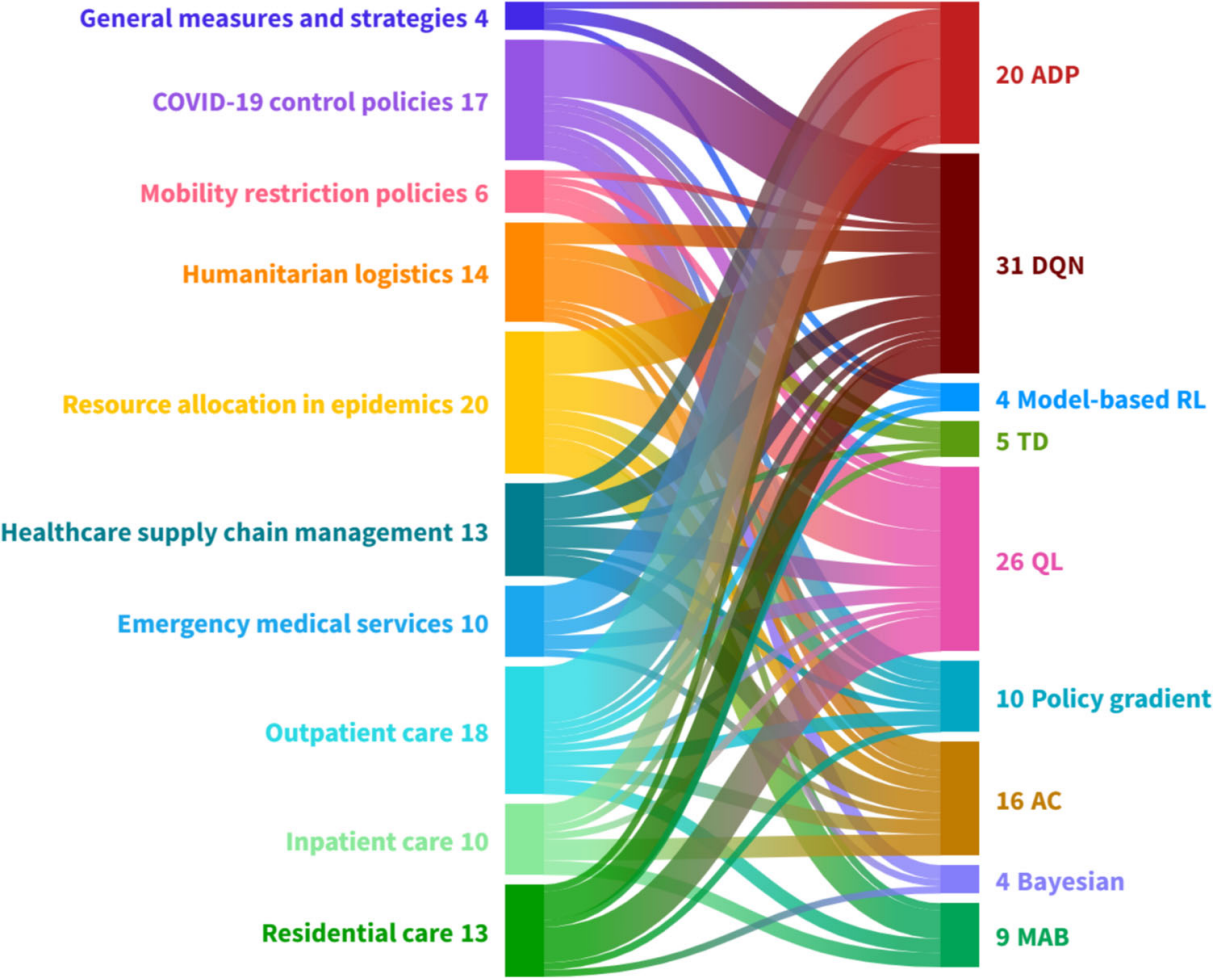
Mapping from applications to learning methods

Our review also reveals that the applications of RL under the microlevel research thrusts have a longer inception period. In addition to the rapid growth observed after 2019, RL applications under the microlevel research thrusts have been consistently developed every year. The majority of these applications utilize ADP to solve the associated MDP, as illustrated in Fig. 2. This is because applications under the microlevel research thrusts, such as surgical scheduling, typically have explicit MDP formulations that allow for the derivation of analytical structures. These characteristics also make ADP a suitable approach. With the advancements in neural networks and deep learning, both ADP and DRL have become viable options for problems under microlevel research thrusts in HOM.

**Fig. 3:**
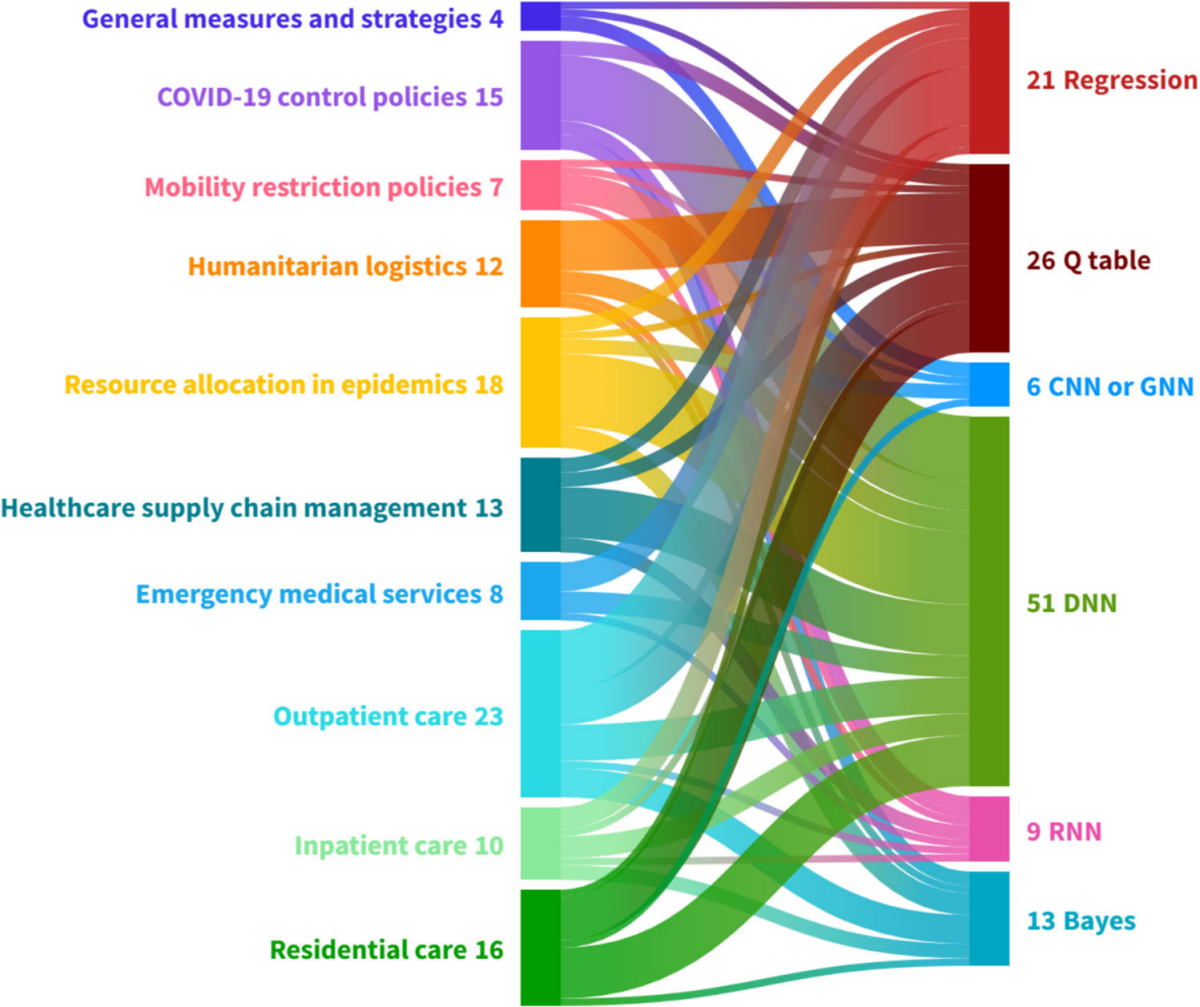
Mapping from applications to approximators

Figures 2 and 3 offer further insights into the popularity of RL methods in different HOM applications.

Figure 2 presents the mapping from HOM applications to RL learning methods. Among the 62 studies reviewed, value-based TD, QL, and DQN are the most popular choices. These methods are particularly prevalent in applications such as COVID-19 control policies, humanitarian logistics, and resource allocation in epidemics. Additionally, more than half of the reviewed applications for residential care implemented QL or DQN. On the other hand, policy-based methods are widely dispersed across all three levels of HOM applications.

Figure 3 presents the mapping from applications to learning approximation methods, which aligns with the results illustrated in Fig. 2. Q table and DNN approximators account for the largest proportion of applications, totaling 73 studies. These approximators correspond to QL and DQN learning methods, respectively. Regression approximators are extensively utilized in EMSs, outpatient care, and inpatient care under the microlevel research thrusts. This is because regression approximators provide an efficient approximation of the value functions of ADP, as shown in Fig. 2. Bayesian inference is employed to estimate the values of actions in MAB frameworks and guide decision-making.

**Fig. 4:**
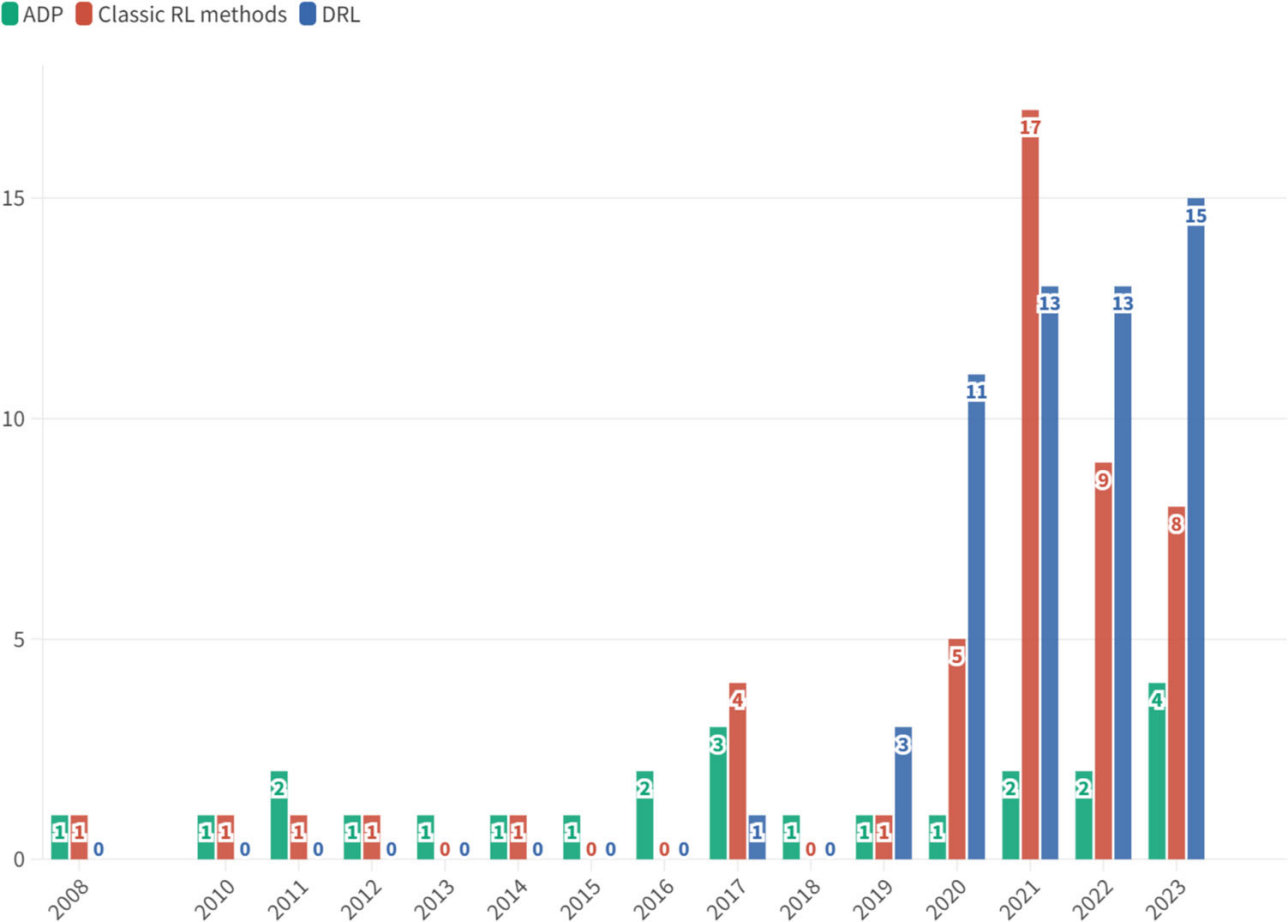
Evolution of RL methodologies used in HOM

Given that the research on RL for HOM falls within the fields of OR and CS, it is interesting to investigate the evolution of methodologies, as discussed in Sect. 2. Figure 4 illustrates this evolution. OR researchers typically develop ADP methods, while classic RL methods such as TD and MAB focus on learning mechanisms rather than neural networks. On the other hand, CS researchers often use DRL methods like DQN and AC with neural networks.

Our analysis reveals that ADP and classic RL methods have been applied for over a decade, with a steady but small number of ADP studies each year. Classic RL methods gained popularity during the COVID-19 pandemic. This trend is consistent with DRL applications, which were first introduced as early as 2017 [219]. Prior to 2017, the amount of research on ADP and classic RL in HOM remained steady, where the two approaches were often used together. However, since then, classic RL and DRL methods have become more dominant, surpassing ADP, especially after 2019.

### Current development

Based on the discussions in Sects. 4 and 5.1, we summarize the current development of effective RL methods in HOM, referring to the key RL settings presented in Sect. 2.3.

One of the most critical discussions is the effectiveness of model-based and model-free RL in HOM. We have observed that model-free RL has been widely applied to macrolevel research thrust, while model-based methods are more applied to microlevel applications. At the macrolevel, the system models usually utilize complicated compartmental models in epidemiology governed by ordinary differential equations [153]. Most reviewed studies tend to rely on the power of “black box" neural networks to learn the system model and find suboptimal policies. This idea is like using complex methods to solve complex problems. Although satisfying results can be produced after sufficient iterations of RL’s experiments and simulations, robustness and interpretability would also be essential for real-world deployments. At the microlevel, the system models usually refer to queueing models [30, 44] or bandit problems [92, 122] under outpatient or inpatient scenarios, where strong theoretical supports are established. In this way, model-based RL can leverage the structures of these models to derive properties of convergence, transitions, or optimal solutions. The robustness, reliability, and interpretability can thus be strong. At the mesolevel, which is in between the macrolevel and microlevel in terms of problem scales and complexity, we have identified some studies that are pursuing model-based robust solutions [177] and realizing interpretable real-world deployments [88]. It emphasizes the importance of interpretable model-based methods when we are transferring research to practice. Therefore, model-free algorithms can be suitable for complex macrolevel HOM problems, where explicit models are not fully available. The success of model-based methods in microlevel applications inspires us to make good use of system models in optimizing decision-making policies. If the system model of the HOM problem is reasonably accurate and theoretically well-defined, model-based RL could be a suitable choice.

The choice of tabular or non-tabular, value-based or policy-based methods for HOM depends on the state and action spaces of specific problems. Given the limitation on problem scales, tabular methods in HOM have only been effectively applied to some routing problems in humanitarian logistics [47, 49]. They provide theoretical foundations for more advanced non-tabular methods and always serve as the benchmark for other RL algorithms’ evaluation in HOM. Non-tabular methods would be required for addressing problems with high-dimensional state space and tractable action space, such as determining macrolevel discrete epidemic controls [144, 147]. Figure 2 also indicates that value-based non-tabular ADP and DQN have been widely applied to HOM applications. If the action space is huge or continuous, such as mesolevel inventory decisions [75, 193], pure value-based methods may fail and policy-based methods (e.g., the AC family) are more appropriate.

Given the fact that most of HOM’s system models are built with simulation techniques, both on-policy and off-policy methods were consistently applied in every research thrust of HOM. As discussed in Sect. 2.3, off-policy methods can be used with existing expert experiences via imitation learning [101], by which the convergence issue is expected to be solved effectively. Further, online algorithms under the realm of MAB are particularly suitable for HOM with dynamic environments, where uncertainty is a main concern. Successful applications include mesolevel resource allocation in epidemics [88, 90] and microlevel resource matching in outpatient [91, 92] and inpatient units [122, 228]. Online learning’s adaptability and interpretability are strengths for solving practical applications [88]. If sample paths for learning are lacking in some HOM applications, offline algorithms with experience replay can sufficiently learn from the limited samples and work out a stable policy. Typical RL methods, like DQN and DDPG, usually combine online and offline methods to achieve adaptability and stability simultaneously. These methods have been utilized in finding macrolevel mobility restriction policy under pandemics, along with the SIHR model [145].

Although the purpose of developing RL approaches is to solve large-scale applications, practical deployment of RL in real-world HOM problems remains a challenge. Most reviewed studies tended to utilize real-world data and simulation-based experiments to benchmark other approaches or real-world experts’ policies. However, only a small number of the studies solved large-scale problems in practice. Under the macrolevel research thrusts, the studies focus on the development of RL for optimal healthcare policies and strategies. In the studies, RL has a superior performance to human experts’ decisions. These studies have built large-scale simulations (e.g., modeling populations of millions of people [121, 130, 139]) and considered high-dimensional state space [121, 133, 134, 146] and action space [134, 146] (e.g., as large as $2.16 \times 10^{59}$ state-action pairs as in [133]). Our review has not identified any practical implementations of healthcare strategies (e.g., lockdown or mobility restriction policies) that solely rely on RL approaches. These macrolevel healthcare strategies are vital to massive stakeholders, and RL solutions are expected to provide references and assistance for the government and decision-makers. Under the mesolevel research thrusts, Bastani et al. [88] have deployed their MAB framework to test kit allocations across 40 Greek borders during pandemics in the summer of 2020. It is an astoundingly impressive large-scale, nationwide, real-world RL in HOM application. The proposed RL approach utilized at most 54,614 passenger locator forms a day, utilizing as many as 185,280 features (i.e., the state space in their problem). Under microlevel research thrusts, D’Aeth et al. [215, 217] optimized a large-scale care prioritization scheme that involves 10 million patients in a case study of England. Their proposed weakly coupled DP had around $15^{10,000,000}$ states and $6^{10,000,000}$ actions. Notably, the authors highlighted the future improvements for real-world implementations.

### Challenges and directions

Based on our scoping review of RL methods in HOM applications, summarized statistics, and current developments, we have identified key insights into the use of RL in HOM. These insights are built upon the advantages of RL in efficiently solving complicated HOM optimization problems.

#### Complexity

HOM applications can be complex. For instance, interactions at the macrolevel and mesolevel, and the integration of emergency care, outpatient care, and inpatient care at the microlevel. Advanced RL algorithms with high-dimensional representations make it possible to solve these complex systems. MARL is a promising framework incorporating multiple homogeneous or heterogeneous RL agents to achieve more precise and complex simulations. MARL has been successfully applied in a number of disaster and emergency response applications in HOM [49, 50, 101, 102, 105, 120, 146, 149, 170, 201]. Another effective modeling approach for complex systems in HOM is the POMDP. In HOM applications, states are often partially observable, and observations can be influenced by unobserved factors such as confounding variables and biased estimations [48, 49, 131]. Therefore, sophisticated algorithms, such as RL with causal inference, are anticipated to address the complexities inherent in the POMDP setting effectively [260]. Under macrolevel and mesolevel research thrusts, hierarchical RL has demonstrated its advantage in efficiently learning and solving large-scale problems [89, 105, 150, 172]. The reviewed three levels are interdependent and need to be considered in concert for integrated care to provide a coordinated and comprehensive healthcare delivery system. Advanced RL algorithms, which efficiently capture the patterns of the complex system with HOM data, will be a strong thrust in this campaign.

#### Adaptability

Given that the HOM applications are always dynamic, the need for flexible and adaptable RL algorithms that can capture the dynamic characteristics of problems and respond to emergency events promptly should be highlighted. Under macrolevel and mesolevel research thrusts, researchers have trained DRL algorithms on various infectious diseases at different stages to ensure their generality [74, 120]. MAB algorithms, known for their scalability, have demonstrated success in real-world epidemic resource allocation [88] and hospital resource matching [92, 122]. Another potential direction is the integration of transfer learning [264] in the RL framework. This approach allows for the utilization of previously learned HOM knowledge from neural networks to handle future similar tasks more effectively. These findings indicate that RL methods with more flexible adaptability will be promising in HOM.

#### Robustness

In the context of HOM, where we need to quantify some metrics related to human lives, robustness is always an essential topic. The estimated HOM-related metrics are typically used as inputs into downstream optimization and decision-making [265]. Due to the uncertainties associated with these estimations, robust optimization [177, 266] can be used to ensure the worst-case performance. However, most RL approaches do not provide theoretical guarantees of the quality of the solutions. To address this, more advanced robust RL methods [74, 120, 141, 187, 267, 268] propelled by control theory show great promise. Safe RL [268] incorporates constraints in the objective function or exploration process and is considered capable of achieving robustness under uncertainty. Another approach is to develop distributionally robust optimization [269] for MDP and benchmark it with RL methods. Optimization paradigms may also involve constraints (e.g., chance-constrained programming and threshold policies [270, 271]) to enhance the robustness of the solutions. Furthermore, there are combinations of optimization and learning [272–274] that accelerate exact combinatorial optimization via RL. In HOM, the need for robustness is consistent with the need for adaptability. It means we need to seek optimal solutions under dynamic and uncertain HOM environments.

#### Interpretability

Communicating effective decisions to human decision-makers is vital in HOM. However, there is a dilemma between using “black box" neural networks [275] to solve complex systems and achieving good interpretability. As a result, some choices, such as MAB algorithms without neural networks, are of greater popularity. These methods approximate value functions using Bayesian or frequentist approaches, providing a level of interpretability. The prevalence of ADP in microlevel applications also highlights the importance of model-based RL, which allows for a deeper understanding of the underlying environments. Multiple selected policy explanation approaches in other fields (mostly visual tasks), such as contrasting rollouts [276], determining critical states [277], utilizing attention mechanisms [278], programmatically interpretable RL [279], explaining through intended outcomes [280], and distal explanations with causal lens [281], can be extended to HOM. These approaches can be integrated into distillation and mimicking paradigms, as discussed in a comprehensive explainable DRL review [165]. Additionally, post hoc techniques can partially explain and inspect “black box" models in DRL, such as the Shapley Additive Explanations [165, 282–284]. Exploring interpretable analysis in DRL will be an interesting and impactful direction for enhancing the practical implementation of decisions in HOM.

#### Validation

Validating the optimal results obtained from RL before deployment in HOM can be a challenging task. Designing an effective measurement of rewards and benchmarking them is not straightforward. One approach is to compare the RL results with exact optimization methods and expert policies. Expert policies, which can serve as “supervisors" in imitation learning [101, 107], can guide and accelerate RL training while also aiding in constructing rewards [285]. In addition, RL performance relies on off-policy evaluation methods [286] as a means of validation, particularly in critical healthcare applications. Causal inference techniques can be used to validate RL decisions [88, 260]. The combination of RL and causal inference in off-policy evaluation has shown great potential [260, 287]. Validation is also closely related to the interpretability of RL [165]. Explicit and interpretable models, as well as model-based methods, have advantages in validating their results. This is because the optimality gap can be theoretically derived, providing a solid foundation for validating the performance of these methods.

#### RL from human feedback

Recently, trendy large language models (LLMs) have highlighted the importance of RLs with human advice [288, 289]. Under the umbrella of human-in-the-loop RL [290], these methods can perform tasks more aligned with human goals by preference-based RL [291] and achieve effective imitation learning [107] or curriculum learning [111]. If the data from human advice are of high quality, the training can be efficient even without the need for massive samples [292]. The interaction between humans and RL can be at different levels depending on who dominates the control of the learning process [293]. RL from human feedback (RLHF) can influence and be applied to every aspect of HOM. The critical states, policies, and rewards of HOM applications can be shaped according to human advice. Critical constraints in HOM summarized by human experts can be integrated into safe RL [268]. The robustness and explorations of RL in HOM can be improved by handling uncertainty and trust regions [294]. RLHF can also substantially help promote the interpretability and validation of RL in HOM [165, 293]. The concept of human-in-the-loop and interoperability are tightly coupled with each other. With RLHF, humans are able to have greater understanding and control over the generated RL policies. Therefore, it is a promising direction for better practical deployment of RL policy in HOM.

#### Real-world implementations

As we have investigated in Sect. 5.2, RL’s limited successful real-world applications in HOM can be attributed to the challenges abovementioned. Modern RL methods have advantages in advancing complex and large-scale HOM applications. While, strong adaptability and robustness are pillars of effective modern RL methods, especially, when tackling emergent practical issues and ensuring the worst-scenario health outcomes. In terms of real-world implementations, interpretability is necessary to explain the optimal policies generated for human stakeholders’ understanding. Rigorous theory and validation of the methodologies and policies are also essential. Therefore, model-based methods with strong interpretability and theoretical performance guarantees are promising. Furthermore, the use of RL in HOM is subject to strict regulatory, ethical, and safety requirements due to the importance of patient health outcomes. RL solutions with more human interactions are expected to make a difference. Only if the challenges of adaptability, robustness, interpretability, and validation are adequately addressed can modern RL methods be implemented in the real world.

## Conclusion

RL is an approach that builds upon MDP for sequential decision-making and aims to address the challenges posed by the curse of dimensionality. Our paper begins with a tutorial on RL methodologies, ranging from MDP to ADP and DRL, followed by a comprehensive scoping review. Our review provides a detailed analysis of RL methodologies and their applications in different domains of HOM, which are classified into macrolevel, mesolevel, and microlevel research thrusts. We analyze the performance of these RL methodologies in HOM. Given the significant impact of the COVID-19 pandemic on the world in recent years, our paper also provides a better understanding of the applications of RL in HOM and how these approaches can improve preparedness for future emergencies. For example, RL has already been implemented in large-scale COVID-19 test kit allocation on Greek borders [88]. Finally, the paper presents statistics on trends, recent developments, and challenges, providing valuable insights into the current state of the field and potential avenues for future research.

Based on our review, we provide the answer to the research questions in Sect. 1:

1. RL methods show great potential in solving complex HOM problems that involve MDP formulations and high dimensionality. Traditional optimization methods often struggle to find exact solutions for such problems in an acceptable time frame, while simple heuristic approaches may result in suboptimal solutions. In this review, RL algorithms have been compared to various benchmarks, including MILP, heuristic methods, and real-world expert policies. The results demonstrate that RL can achieve good performance in terms of both solution effectiveness and computational efficiency. Although RL training time can be long as problem scales grow, RL has the ability to learn problem-specific features during training and can be transferred to similar situations through transfer learning. Additionally, imitation learning can provide a “warm start" for RL training. These characteristics and techniques make RL a suitable approach for tackling complex HOM problems.
2. Our comprehensive investigation of RL methods applied in HOM reveals that ADP and DRL approaches are among the most popular methods. However, the choice of the most suitable and effective RL methods depends on the specific HOM problems at hand. For highly complex HOM models, neural network approximators are expected to be effective in achieving desired outcomes. Conversely, when a model has an explicit planning framework, model-based methods can enhance robustness, interpretability, and validation in the face of uncertainty. According to our review, it is challenging to simultaneously achieve highly complex RL with “black box" approximators and model-based RL with strong interpretability and theoretical performance guarantee. Interpretable RL in HOM is, therefore, one of the most promising future directions.
3. In Sect. 5, we have discussed the recent developments, challenges, and potential future directions for RL in HOM. Since the RL’s high-dimensional representation can partly address the complexity in HOM applications, it is believed that developing RL for HOM purposes with a focus on developing adaptability, robustness, interpretability, validation, and RLHF holds promise. These five directions will enable better preparation and real-world large-scale solutions for future HOM problems.

In conclusion, RL for HOM is an emerging field with significant potential. The effective integration of RL methodologies and application modeling techniques is crucial for achieving optimal results. The synergy between these two phases holds great promise for advancing the field of HOM.

## Data Availability

The authors confirm that the data supporting the findings of this study are available within the article.
